# Early Main Group Metal Catalysts for Imine Hydrosilylation

**DOI:** 10.1002/chem.201904148

**Published:** 2019-11-18

**Authors:** Holger Elsen, Christian Fischer, Christian Knüpfer, Ana Escalona, Sjoerd Harder

**Affiliations:** ^1^ Inorganic and Organometallic Chemistry Friedrich-Alexander-Universität Erlangen-Nürnberg Egerlandstraße 1 91058 Erlangen Germany

**Keywords:** alkaline earth metals, density functional calculations, hydrosilylation, imines, potassium

## Abstract

The efficient catalytic reduction of imines with phenylsilane is achieved by using the potassium, calcium and strontium based catalysts [(DMAT)K (THF)]_∞_, (DMAT)_2_Ca⋅(THF)_2_ and (DMAT)_2_Sr⋅(THF)_2_ (DMAT=2‐dimethylamino‐α‐trimethylsilylbenzyl). Eight different aldimines and the ketimine Ph_2_C=NPh could be successfully reduced by PhSiH_3_ at temperatures between 25–60 °C with catalyst loadings down to 2.5 mol %. Also, simple amides like KN(SiMe_3_)_2_ or Ae[N(SiMe_3_)_2_]_2_ (Ae=Ca, Sr, Ba) catalyze this reaction. Activities increase with metal size. For most substrates the activity increases along the row K<Ca<Sr<Ba. Fastest conversion was found for imines with alkyl substituents at N and aryl rings at C, for example, PhC(H)=N*t*Bu, while *t*BuC(H)=N*t*Bu or PhC(H)=NPh react much slower. Reasonable functional group tolerance is observed. The proposed metal hydride mechanism is supported by stoichiometric reactions using a catalyst model system, isolation of intermediates and DFT calculations.

## Graphical abstract

**First** ***s*** **‐block metal** catalysts for imine hydrosilylation are reported. The mechanism is confirmed by stoichiometric reactions, intermediate isolation and DFT calculations.

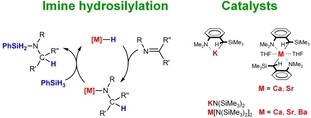

## Introduction

Due to the high demand of amines in the field of pharmaceuticals and agrochemicals, the catalytic conversion of imines to amines has become an important field of research. Although a large part of this research focuses on direct imine hydrogenation, the reduction of the C=N bond with an easy to handle and safe silane has become an important alternative.^1^ While late transition metal catalysts are well‐established for imine hydrosilylation,^2^, ^3^ the focus of current research is shifted towards cheaper more environmentally and benign metals.^4^, ^5^ In 2006, Yun et al. published the first catalytic hydrosilylation of imines using a zinc based catalyst.^6^, ^7^ There is, however, little known in literature about the catalytic hydrosilylation of C=N bonds using *s*‐block metals.

**Scheme 1:**
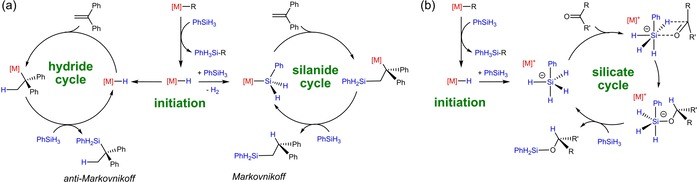
(a) Proposed mechanisms for the catalytic alkene hydrosilylation with K, Ca or Sr catalysts. Metal or solvent choices determine the mechanism and regioselectivity. (b) Proposed silicate cycle for catalytic ketone hydrosilylation.

In 2006, we introduced alkene hydrosilylation using potassium‐, calcium‐ and strontium‐based catalysts (Scheme 1 a) and found that the regioselectivity in some cases can be tuned either by metal or solvent choice.^8^ Formation of the *anti*‐Markovnikoff product was explained by the hydride cycle whereas for the Markovnikoff product a silanide cycle is operative. Since especially the metals potassium and calcium hold the advantage that they are both non‐toxic as well as inexpensive, they make for excellent alternatives to noble‐metal catalysts. Asgari et al. reported the hydrosilylation of activated alkenes using simple *s*‐block metal bases such as KOH or alcoholates.^9^ This, however, has the drawback that the high degree of regiocontrol observed by Harder is lost. In 2008 we extended our work with group 2 metal catalysts for ketone hydrosilylation.^10^ Especially for ketone substrates like RCH_2_C(O)R′, which easily form enolates by deprotonation, a mismatch was found between products obtained from stoichiometric reactions with a calcium hydride complex and those obtained in a catalytic hydrosilylation regime. On this basis we proposed a mechanism that circumvents formation of a metal hydride intermediate and instead it was suggested that the hydride is transferred directly from a silicate intermediate (Scheme 1 b). Later reports on ketone hydrosilylation with simple potassium alkoxide catalysts support this hypothesis and propose a similar mechanism.^11^

Although early main‐group‐metal catalysis has made substantial progress,^12^ it is surprising that hitherto imine hydrosilylation has not been reported. The closest to imine hydrosilylation is the recently described catalytic dearomatization of the C=N bond in pyridine by 1,2‐selective hydrosilylation using a calcium catalyst.^13^ In contrast, magnesium catalysts showed no activity in pyridine hydrosilylation but performed well in its hydroboration.^14^, ^15^ In this work we present the first investigations on imine hydrosilylation using early main group metal catalysts. We optimize the reaction conditions, discuss the substrate scope and also provide experimental as well as calculational evidence for a potential mechanism.

## Results and Discussion

**Figure 1:**
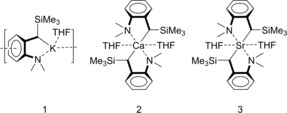
Catalysts for the hydrosilylation of imines.

The hydrosilylation of imines has been investigated using the following complexes as catalysts (Figure 1): [(DMAT)K (THF)]_∞_ (**1**), (DMAT)_2_Ca⋅(THF)_2_ (**2**) and (DMAT)_2_Sr⋅(THF)_2_ (**3**). These complexes, which are already well established catalysts for ketone or alkene hydrosilylation,^8^, ^10^ contain the 2‐dimethylamino‐α‐trimethylsilylbenzyl anion (DMAT) in which the negative charge is stabilized through negative hyperconjugation by the Me_3_Si substituent as well as by delocalization in the aromatic ring. The *ortho*‐Me_2_N‐substituent provides stabilization by metal coordination. The Ca and Sr complexes are highly stable molecular entities that do not deprotonate THF and are very well soluble in aromatic solvents like benzene. However, the K complex forms a coordination polymer in the solid state and shows only limited solubility in aromatic solvents.

All catalytic reactions were carried out in C_6_D_6_ and were directly monitored by ^1^H NMR (Table 1). Substrate conversion has been determined by integration of characteristic reactant and product signals. The hydrosilylation of a variety of imines (**I**–**IX**) with PhSiH_3_ catalyzed by **1**–**3** has been investigated. In the absence of a catalyst, the imine substrates did not react with phenylsilane. In most cases, the catalytic imine hydrosilylation proceeded cleanly and no side products were observed in ^1^H NMR or GC‐MS analysis.

**Table 1:** Catalytic imine hydrosilylation.^[a]^

Entry	Catalyst	Loading [mol %]	Substrate	*T* [°C]	*t*	Conv. [%]
1	(DMAT)K⋅(THF)	2.5	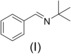	25	24 h	10
2	(DMAT)K⋅(THF)	5		25	24 h	80
3	(DMAT)K⋅(THF)	5		60	19 h	99
4	(DMAT)K⋅(THF)^[d]^	5		25	20 min	99
5	(DMAT)_2_Ca⋅(THF)_2_	5		25	15 min	99
6	(DMAT)_2_Ca⋅(THF)_2_	5		60	5 min	99
7	(DMAT)_2_Ca⋅(THF)_2_^[d]^	5		25	8 h	99
8	(DMAT)_2_Sr⋅(THF)_2_	2.5		25	5 min	99
9	(DMAT)_2_Sr⋅(THF)_2_	1		60	24 h	traces
10	(DMAT)_2_Sr⋅(THF)_2_^[d]^	5		25	1.5 h	99
11	[CaH(BDI)⋅(THF)]_2_	2.5		25	4 h	99
12	[CaH(BDI)]_2_	2.5		25	15 min	99
13	KN(SiMe_3_)_2_	5		25	24 h	77
14	Ca[N(SiMe_3_)_2_]_2_	5		25	1.75 h	99
15	Sr[N(SiMe_3_)_2_]_2_	5		25	15 min	99
16	Ba[N(SiMe_3_)_2_]_2_	5		25	10 min	99
17	(DMAT)K⋅(THF)	2.5	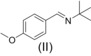	25	24 h	12
18	(DMAT)_2_Ca⋅(THF)_2_	2.5		25	80 min	99
19	(DMAT)_2_Sr⋅(THF)_2_	2.5		25	5 min	99
20	(DMAT)K⋅(THF)	2.5	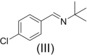	25	24 h	21
21	(DMAT)_2_Ca⋅(THF)_2_	2.5		25	6 h	99
22	(DMAT)_2_Sr⋅(THF)_2_	2.5		25	20 min	99
23	(DMAT)K⋅(THF)	2.5	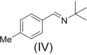	25	24 h	43
24	(DMAT)_2_Ca⋅(THF)_2_	2.5		25	24 h	traces
25	(DMAT)_2_Ca⋅(THF)_2_	5		25	40 min	99
26	(DMAT)_2_Sr⋅(THF)_2_	2.5		25	24 h	89
27	(DMAT)K⋅(THF)	2.5	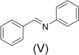	25	5 min	99
28	(DMAT)_2_Ca⋅(THF)_2_	2.5		25	5.5 h	99
29	(DMAT)_2_Sr⋅(THF)_2_	2.5		25	70 min	99
30	(DMAT)_2_Sr⋅(THF)_2_	2.5		60	10 min	99
31	(DMAT)K⋅(THF)	2.5	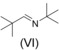	25	24 h	0
32	(DMAT)K⋅(THF)	5		60	24 h	traces
33	(DMAT)_2_Ca⋅(THF)_2_	2.5		25	24 h	89
34	(DMAT)_2_Sr⋅(THF)_2_	2.5		25	12.5 h	99
35	(DMAT)_2_Sr⋅(THF)_2_	5		25	1.5 h	99
36	(DMAT)K⋅(THF)	5	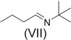	25	24 h	traces
37	[(DMAT)_2_Ca⋅(THF)_2_	2.5		25	35 min	99^[c]^
38	[(DMAT)_2_Sr⋅(THF)_2_	2.5		25	15 min	99^[c]^
39	(DMAT)K⋅(THF)	2.5	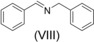	25	24 h	84
40	(DMAT)_2_Ca⋅(THF)_2_	2.5		25	10 min	99
41	(DMAT)_2_Sr⋅(THF)_2_	2.5		25	5 min	99^[b]^
42	(DMAT)K⋅(THF)	5	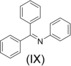	60	21 h	99
43	(DMAT)_2_Ca⋅(THF)_2_	5		60	24 h	50
44	(DMAT)_2_Sr⋅(THF)_2_	5		60	21 h	99
45	(DMAT)_2_Sr⋅(THF)_2_	2.5		60	24 h	44

[a] Reaction times for >99 % conversion (determined by ^1^H NMR) were optimized in 5 minute steps. For slower reactions the conversion after 24 hours is given. [b] Multiple addition of imine to PhSiH_3_ observed in GC−MS. [c] Isomerization of the double bond and double silylation observed in GC−MS. [d] in 500 μL [D_8_]THF.

In a first series of experiments the benchmark imine substrate *E*‐PhC(H)=N*t*Bu (**I**) was converted with PhSiH_3_ in order to optimize the reaction conditions. The K catalyst **1** gave at room temperature hardly any conversion (entry 1). Since this may find its origin in the poor solubility of **1** in C_6_D_6_, a small amount of THF (ca. 2.5 equiv) was added but this barely gave any improvement. An increase of the catalyst loading from 2.5 to 5 mol % catalyst loading led to a considerable rise in conversion (entry 2) and, when heated to 60 °C, full conversion is achieved in 19 hours (entry 3). In stark contrast, the Ca catalyst (DMAT)_2_Ca⋅(THF)_2_ (**2**) converted imine **I** significantly faster, both at room temperature and 60 °C (entries 5–6). The more polar and reactive Sr catalyst **3** was, as expected, clearly the fastest catalyst: at room temperature imine **I** was fully hydrosilylated by PhSiH_3_ within 5 minutes using only a catalyst loading of 2.5 mol % (entry 8). Further lowering of the catalyst loading, however, led only to traces of product, even at 60 °C (entry 9). It is noteworthy that the simple amide complexes M[N(SiMe_3_)_2_]_*n*_ (M=K (*n*=1), Ca, Sr, Ba (*n*=2)) were also found to be catalytically active (entries 13–16) with activities increasing along the row K<Ca<Sr<Ba. Conversions with Ca[N(SiMe_3_)_2_]_2_ or Sr[N(SiMe_3_)_2_]_2_ are clearly slower than those with the corresponding DMAT complexes **2** and **3** but that of the Ba[N(SiMe_3_)_2_]_2_ catalyst is *on par* with that of Sr catalyst **3**.

Switching from benzene to the more polar solvent THF led to a drastic increase of the catalytic performance of the K catalyst (entry 4). This may be explained by its much better solubility in THF. In contrast to this, both the Ca and Sr catalysts need in THF significantly longer reaction times for imine hydrosilylation (entries 7 and 10). Apparently, the Lewis acidity of the metal is much more important in group 2 metal catalysis than for the potassium‐catalyzed reaction. Thus, THF competes with the imine for precoordination and substrate activation.

The influence of the silane on the conversion rates was also investigated. Using Ca catalyst **2** (5 mol %) the benchmark imine **I** was at room temperature reduced by PhSiH_3_ within 15 minutes (entry 5). With the secondary silane Ph_2_SiH_2_ after 24 hours only 9 % conversion was observed. While the tertiary silane Ph_3_SiH gave after 24 hours only traces of the hydrosilylation product, silane (EtO)_3_SiH did not yield any conversion at all. For this reason, further investigations on the scope of imines have been performed with PhSiH_3_.

Functional group tolerance has been investigated for the imines *para*‐X‐PhC(H)=N*t*Bu in which X=MeO (**II**), Cl^III^ or Me^IV^. Introduction of a *para*‐substituent in the phenyl ring generally increases reaction times. The methoxy substituent (imine **II**) only exhibited a significant effect for the Ca catalyst increasing the necessary reaction time to 80 minutes (entry 18). A chloro substituent in the *para*‐position (imine **IV**) lowers activities for both, the Ca and Sr catalysts (entries 21–22). Introduction of a methyl group (imine **V**) led only to formation of traces of product for the Ca catalyst (entry 24) but increasing the catalyst loading from 2.5 to 5 mol % gave full conversion within 40 minutes (entry 25). The Sr catalyst, which generally shows best activities, only gave 89 % conversion after 24 hours (entry 26). These results demonstrate that these catalysts show a fair tolerance towards ether, Cl or benzylic functional groups. However, the most challenging substrate with a NO_2_‐substituent in *para*‐position did not give conversion with any of the catalysts. ^1^H NMR investigation revealed that this is due to a side reaction with the catalyst, resulting in a black reaction mixture and broadened proton resonances.

Substitution of the N‐*t*Bu group in **I** for a phenyl ring (substrate **II**) led to a significant increase in activity for the K catalyst (**1**) giving already at room temperature full conversion within 5 minutes (entry 27). Interestingly, the Ca (**2**) and Sr (**3**) catalysts, which were highly active for imine **I**, needed longer reaction times for full conversion (entries 28–30).

**Scheme 2:**
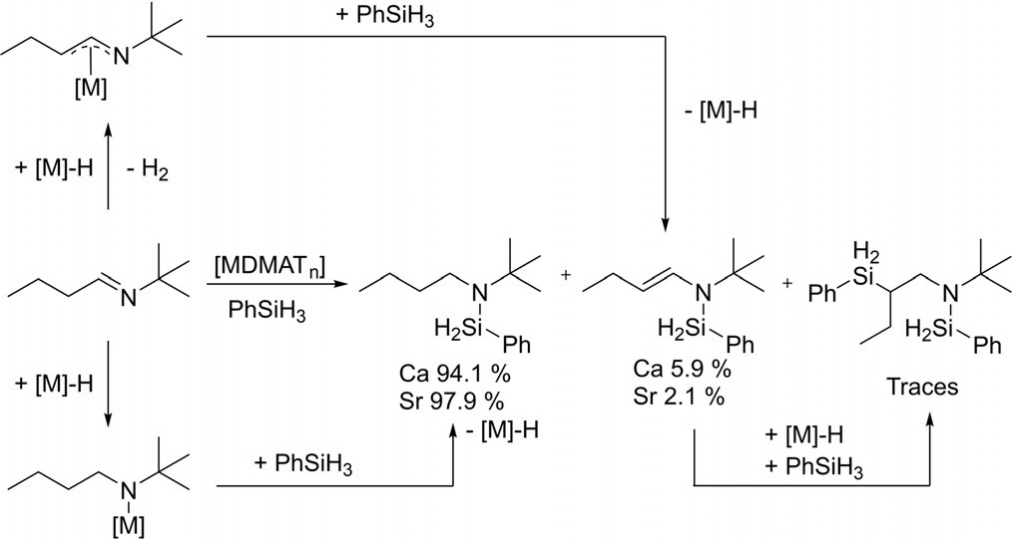
Hydrosilylation of *n*PrC(H)=N*t*Bu (**VII**) and dehydrogenative silylation accompanied by double bond isomerization. The latter likely proceeds through an aza‐allyl intermediate.

We tested imines with aliphatic substituents on C as well as N: *t*BuC(H)=N*t*Bu (**VI**) and *n*PrC(H)=N*t*Bu (**VII**). For **VI** much lower conversion rates have been found. The K catalyst gave even with higher catalyst loadings and raised temperatures only traces of product (entries 31–32) but the more active Ca and Sr catalysts gave at 2.5 mol % catalyst loading at room temperature 89 % conversion in 24 hours and full conversion in 12.5 hours, respectively (entries 33–34). For imine **VII**, with a primary *n*Pr group at C instead of a tertiary *t*Bu group, conversion was found to be much faster (entries 37–38). In this case not only the hydrosilylation product was isolated but also significant quantities of side‐products have been identified. The formation of these species likely proceeds through deprotonation instead of addition (Scheme 2).

The imine PhC(H)=NBn (**VIII**) gave a significant increase in activity when compared to conversion of PhC(H)=N*t*Bu (**I**) (entries 39–41) which becomes most apparent for the K catalyst. Imine **VIII** was the only substrate that showed multiple addition to PhSiH_3_ (entry 41). The most active Sr catalyst gave besides the expected hydrosilylation product (Bn_2_N)SiH_2_Ph also (Bn_2_N)_2_Si(H)Ph and using GC‐MS analysis even traces of (Bn_2_N)_3_SiPh could be detected. These multiple additions to PhSiH_3_ are likely related to the much lower demand for space of the primary benzyl group at N (imine **VIII**) compared to the tertiary *t*Bu substituent (imine **I**). For imine **VIII**, the reaction mixture turned red immediately after addition of the imine, most likely due to deprotonation of the CH_2_ group, creating a benzylic anion with an extended delocalized π‐system. Since dehydrosilylation products could not be detected, this is only a minor side reaction.

Although all substrates **I‐**‐**VIII** are aldimines, our catalysts were also found to be active in the much more challenging hydrosilylation of a ketimine. The ketimine Ph_2_C=NPh (**IX**) did not react at room temperature but at 60 °C both the K and the Sr catalyst performed similar, giving full conversion after 21 hours (entries 42 and 44). In contrast, the Ca catalyst only gave half conversion after 24 hours (entry 43).

Data in Table 1 led to conclude that aryl substituents on the imine C and alkyl substituents on the imine N are most favorable for efficient conversion. The aryl substituent at C activates the C=N bond by conjugation, while an electron‐releasing alkyl group at N makes the amide intermediate much more nucleophilic when compared to a N−Ph substituent which stabilizes the amide anion by delocalization.

We propose that imine hydrosilylation proceeds via a metal hydride mechanism similar to that shown in Scheme 1 a. Reaction of the precatalyst **1**, **2** or **3** with PhSiH_3_ generates a metal hydride species. These might either be nano‐clusters of saline [MH_2_]_*n*_ or mixed DMAT/hydride clusters of general formula [(DMAT)_*x*_CaH_*y*_]_*n*_ (*x*+*y=*2). All attempts to isolate such species by crystallization failed but for the Ae[N(SiMe_3_)_2_]_2_ catalysts such mixed amide/hydride clusters have been characterized by crystal structure determination.^16^, ^17^

**Figure 2:**
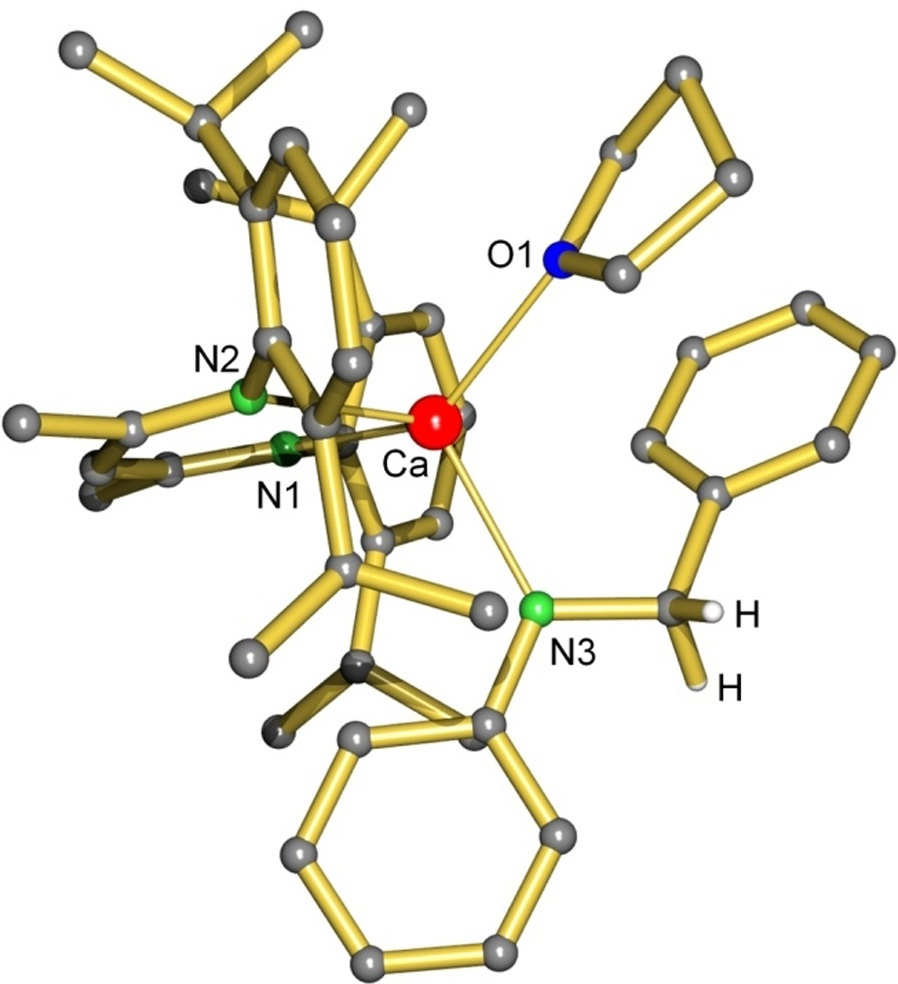
Crystal structure of the intermediate (^DIPP^BDI)Ca[N(Ph)CH_2_Ph]⋅(THF). H atoms partially omitted for clarity. Selected bond lengths (Å): Ca−N1 2.356(1), Ca−N2 2.338(1), Ca−N3 2.297(1), Ca−O1 2.371(1), N3−CH2 1.450(2).

In order to support the hypothetical metal hydride mechanism we reacted the well‐defined Ca hydride complex [(^DIPP^BDI)CaH⋅(THF)]_2_ 18, 19 (^DIPP^BDI=HC[C(Me)=N‐DIPP]_2_; DIPP=2,6‐di*iso*propylphenyl) stepwise with imine PhC(H)=N*t*Bu (**I**) and subsequently with PhSiH_3_. Complex [(^DIPP^BDI)CaH⋅(THF)]_2_, which is well soluble in C_6_D_6_, reacted at 60 °C with two equivalents of **I**. This led to immediate disappearance of the characteristic resonance for the hydride at *δ*=4.79 ppm while a new signal at *δ*=4.13 ppm can be assigned to the CH_2_ group of the reduced imine: PhCH_2_(*t*Bu)N^−^. Although we have not been able to isolate this species, the intermediate could be crystallized when using imine PhC(H)=NPh (**V**). The crystal structure of (^DIPP^BDI)Ca[N(Ph)CH_2_Ph]⋅(THF) was determined by X‐ray diffraction. It shows a Ca center that is chelated by the ^DIPP^BDI ligand and is further bound to PhCH_2_(Ph)N^−^ and one THF ligand (Figure 2). The former C=N double bond now is in the typical range of a C−N single bond with 1.450(1) Å.^20^

Subsequent addition of one equivalent of PhSiH_3_ led for both intermediate amide complexes, that is, that with PhCH_2_(*t*Bu)N^−^ or PhCH_2_(Ph)N^−^, after reaction at 60 °C to formation of the expected hydrosilylation product and the Ca hydride complex [(^DIPP^BDI)CaH⋅(THF)]_2_. This stoichiometric cycle could be repeated at least twice by renewed addition of imine and PhSiH_3_, representing strong evidence that imine hydrosilylation is catalyzed by metal hydride catalysts. Indeed, removing the volatiles from the solution and recrystallization from pentane led to recovery of crystalline [(^DIPP^BDI)CaH⋅(THF)]_2_. The catalytic activity of [(^DIPP^BDI)CaH⋅(THF)]_2_ is somewhat lower than that of (DMAT)_2_Ca⋅(THF)_2_ (*cf*. entries 11 and 5) but the more reactive THF‐free complex [(^DIPP^BDI)CaH]_2_ 21 performs similarly (entry 12). Although there is ample evidence that the metal hydride species is the catalyst, commercially available KH and CaH_2_ did not show any catalytic activity in imine hydrosilylation. This is, however, most likely due to their insolubility in C_6_D_6_.

**Scheme 3:**
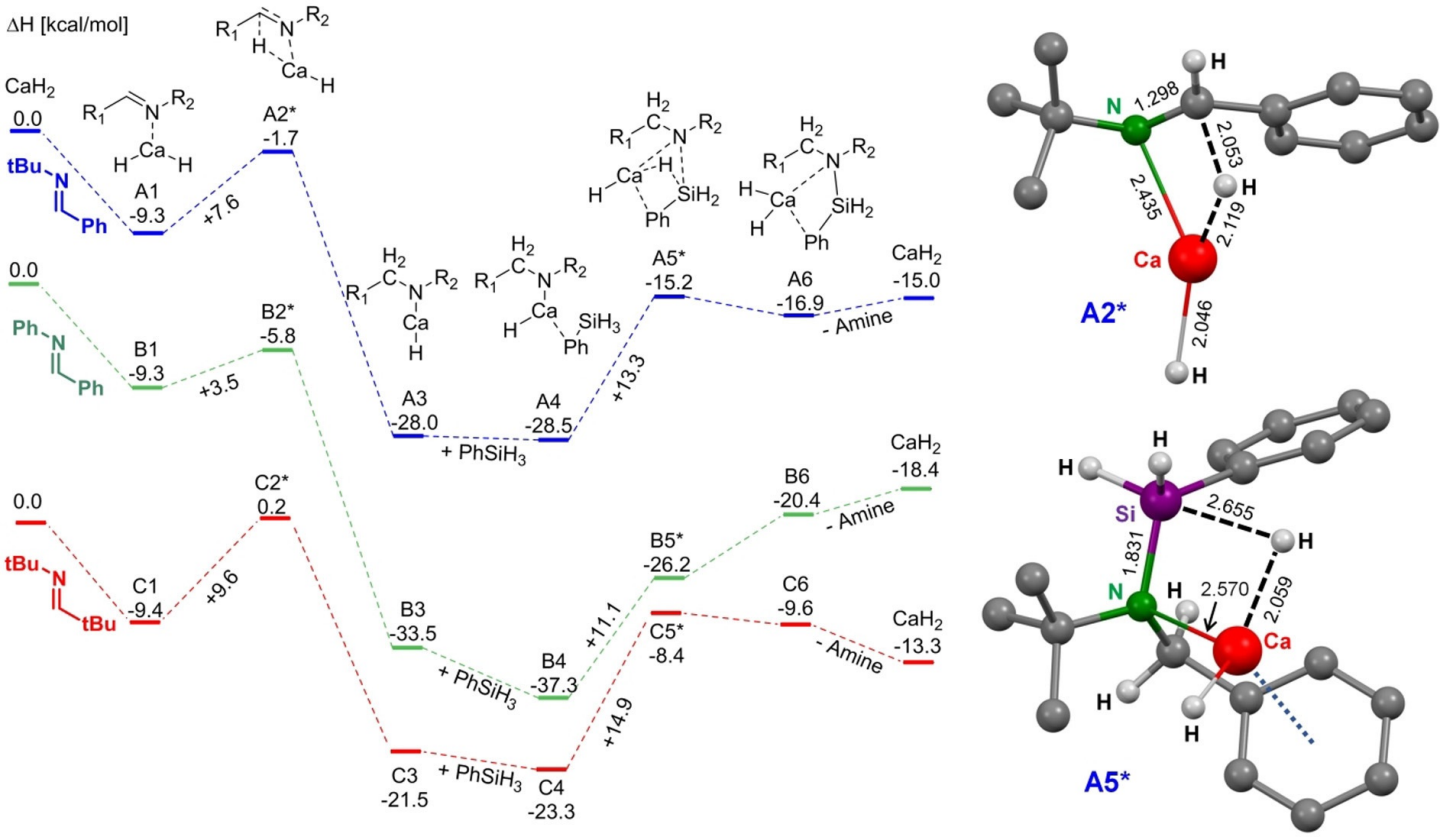
Enthalpy for the catalytic hydrosilylation of imines **I** (blue), **V** (green) and **VI** (red) by CaH_2_ on a B3PW91/6‐311++G** level of theory (PCM=benzene) and structures of the transition states A2* and A5*(some H atoms have been omitted for clarity; selected bond lengths given in Å).

The proposed metal hydride cycle has been modelled by DFT calculations using CaH_2_ as a model catalyst and *E*‐PhC(H)=N*t*Bu (**I**) as the imine substrate (Scheme 3). Calculations have been performed at the B3PW91/6‐311++G** level of theory.^22^, ^23^, ^24^, ^25^ Since the coordination sphere of the metal in CaH_2_ is hardly saturated, gas phase reactions show interaction energies that are strongly overestimated. For that reason, a more realistic reaction profile with solvent correction in benzene using the PCM method has been calculated.

Imine coordination is clearly exothermic (**A1**, −9.3 kcal mol^−1^) and the subsequent transition state for insertion (A2*) is only 7.6 kcal mol^−1^ higher in energy and should be considered early: the Ca−H and C=N bonds are hardly elongated (Scheme 3). The transformation of the metal hydride into a metal amide species is quite exothermic (**A3**, −28.0 kcal mol^−1^). The second step, nucleophilic attack of the metal amide at PhSiH_3_ to give amide‐hydride exchange, clearly has a higher energy barrier (**A4**→A5*) and this rate‐limiting step needs 13.3 kcal mol^−1^. Transition state A5* is stabilized by a Ca⋅⋅⋅Ph interaction with the imine (Scheme 3). The last step, in which CaH_2_ is formed again (**A6**, **A7**), is clearly endothermic but the highly reactive CaH_2_ species is immediately trapped by addition to the imine. The resting state in the catalytic cycle is the Ca amide complex **A3** and the total imine hydrosilylation reaction is exothermic by −15.0 kcal mol^−1^.

In order to rationalize the different effects of alkyl or aryl substituents at the imine C or N atom, also the mechanisms for hydrosilylation of *t*BuC(H)=N*t*Bu and PhC(H)=NPh have been evaluated by DFT methods (Scheme 3). While imine coordination to CaH_2_ is equally exothermic for both substrates (**B1**, 9.3 kcal mol^−1^ & **C1**, 9.4 kcal mol^−1^), the transition state for the reduction to the corresponding amide shows that PhC(H)=NPh is the most readily reduced. The activation barrier is only 3.5 kcal mol^−1^ (B2*) while the aliphatic *t*BuC(H)=N*t*Bu imine shows the highest activation barrier with 9.6 kcal mol^−1^ (C2*). This can be rationalized by the fact that aromatic substituents activate the imine C=N bond by conjugation. The energy gained by amide formation is strongly exothermic for both imines with −33.5 kcal mol^−1^ (**B3**) and −21.5 kcal mol^−1^ (**C3**), respectively. The much higher stability of the PhCH_2_(Ph)N^−^ anion can be explained by delocalization of negative charge in the Ph ring. Coordination of PhSiH_3_ to give complexes **B4** and **C4** leads to energy gain of −1.8 and −3.8 kcal mol^−1^, respectively, and is therefore more exothermic than for formation of **A4** (−0.5 kcal mol^−1^). The amide‐hydride exchange at calcium is again the rate‐limiting step with an activation barrier of +11.1 kcal mol^−1^ and +14.9 kcal mol^−1^ for transition states B5* and C5*, respectively. Due to the reduced nucleophilicity of PhCH_2_(Ph)N^−^ compared to *t*BuCH_2_(*t*Bu)N^−^ or *t*BuCH_2_(*t*Bu)N^−^, it was expected that the activation energy for B5* is higher than for C5* or A5*. This would also be in accordance with the slower hydrosilylation of PhC(H)=NPh compared to PhC(H)=N*t*Bu. In contrast, however, formation of B5* has the lowest activation energy. The low reactivity of PhC(H)=NPh may be explained by the very high stability of **B4** (compared to **C4** and **A4**) and the fact that product elimination from the catalyst is endothermic. The conversion of the Ca amide intermediates **A4**, **B4** and **C4** into CaH_2_ and the product is most endothermic for **B4** (+18.9 kcal mol^−1^); *cf*. **A4** (+13.5 kcal mol^−1^) and **C4** (+10.0 kcal mol^−1^). Route **B** is also the only pathway for which the formation of the CaH_2_⋅(product) complex **B6** is endothermic. This may explain why PhC(H)=N*t*Bu (**I**) is more readily hydrosilylated than PhC(H)=NPh (**V**) even though the latter has the lowest activation barriers of the three imines. The overall hydrosilylation reaction is exothermic by −18.4 kcal mol^−1^ for PhC(H)=NPh (**V**) and −13.3 kcal mol^−1^ for *t*BuC(H)=N*t*Bu **VI**.

## Conclusions

The first *s*‐block metal catalysts for imine hydrosilylation have been introduced. The three benzyl complexes (DMAT)K⋅(THF), (DMAT)_2_Ca⋅(THF)_2_ and (DMAT)_2_Sr⋅(THF)_2_ catalyze hydrosilylation of eight different aldimines and one ketimine with PhSiH_3_. In addition, it could also be shown that simple amide complexes like Ae[N(SiMe_3_)_2_]_2_ (Ae=Ca, Sr, Ba) catalyze this reaction, albeit it slightly slower than the benzyl‐based catalysts. Generally, an increase in activity was found along the row K<Ca<Sr<Ba. In some cases, full conversion was reached with the Sr catalyst (2.5 mol %) within five minutes at room temperature. The fastest conversion was found for imines with alkyl substituents at N and aryl rings at C, for example, PhC(H)=N*t*Bu. Variation of substituents in the *para*‐position of the Ph ring (Me, Cl, MeO) is possible and indicates a reasonable functional group tolerance. Imines like *t*BuC(H)=N*t*Bu or PhC(H)=NPh react much slower. It is favorable when the C=N bond is conjugated with an aryl ring while a Ph group at N slows down conversion. This can be explained by the catalytic cycle which involves: (i) generation of a metal hydride intermediate, (ii) addition of the metal hydride to the imine to give an amide and (iii) conversion of the amide by PhSiH_3_ into the hydrosilylation product and the metal hydride species. A Ph group at N stabilizes the intermediate amide complex by resonance making it less nucleophilic. The proposed metal hydride mechanism is supported by stoichiometric reactions and isolation of reaction intermediates. It is also confirmed by DFT calculations. Depending on the substitution pattern, activation enthalpies between 3.5 and 9.6 kcal mol^−1^ for CaH_2_ to imine addition have been estimated. The second step, metal amide to hydride conversion, is the rate‐determining step with activation enthalpies in the 11.1–14.9 kcal mol^−1^. The results presented in here broaden the scope of early main group metal catalysts.

## Experimental Section

All experiments were carried out using standard Schlenk techniques or a glove box (MBraun, Labmaster SP) and freshly dried solvents. THF (THF AnalaR Normapur, VWR) was dried over molecular sieves (3 Å) and distilled from sodium. All other solvents were degassed with nitrogen, dried over activated aluminum oxide (Innovative Technology, Pure Solv 400‐4‐MD, Solvent Purification System) and stored over molecular sieves (3 Å) under inert atmosphere. Starting materials were used as delivered unless noted otherwise. Imine PhC(H)=N*t*Bu was purchased from Sigma–Aldrich, stirred over CaH_2_ and distilled prior to use. The following compounds were prepared according to literature procedures: catalysts **1**–**3**,^26^ [(^DIPP^BDI)CaH⋅(THF)]_2_,^19^ [(^DIPP^BDI)CaH]_2_,^21^ [CaN(SiMe_3_)_2_]_2_, [SrN(SiMe_3_)_2_]_2_ and [BaN(SiMe_3_)_2_]_2_.^27^

Imine substrates were prepared according to the respective literature procedures: PhC(H)=NPh,^28^ *t*BuC(H)=N*t*Bu,^29^ *p*‐Cl‐PhC(H)=N*t*Bu,^30^ *p*‐MeO‐PhC(H)=N*t*Bu,^30^ PhC(Me)=N*t*Bu,^31^ PhCH=NCH_2_Ph,^32^ *n*PrC(H)=N*t*Bu,^33^ Ph_2_CH=NPh.^34^

### Catalytic imine hydrosilylation

All substrates were dried by stirring over freshly powdered CaH_2_ at 60 °C. Deuterated benzene was dried statically over molecular sieves (3 Å). All catalytic experiments were prepared in an MBraun LabMaster Glovebox filled with dry N_2_. The catalyst (0.0125 or 0.0250 mmol) was placed in a J‐Young NMR tube and the imine (0.500 mmol) was added. The mixture was dissolved in 500 μL C_6_D_6_ and PhSiH_3_ (81.1 mg, 92.5 μL, 0.750 mmol). Conversions were determined via ^1^H NMR spectroscopy by integration of characteristic signals.

### Synthesis of complex (^DIPP^BDI)Ca[N(Ph)CH_2_Ph]⋅(THF)

A solution of complex [(^DIPP^BDI)CaH⋅(THF)]_2_ (53.0 mg, 0.0499 mmol) and the imine PhC(H)=NPh (**V**) (18.1 mg, 0.0998 mmol) was heated in 525 μL of C_6_D_6_ at 60 °C for three days. The solution was concentrated under reduced pressure to approximately half its original volume and allowed to stand for three days. The product was received in the form of colorless crystals. Yield 57.3 mg, 0.0805 mmol, 81 %. Elemental analysis: calcd: C 78.71 %, N 5.57 %, H 8.57 %. Found: C 79.04 %, N 5.32 %, H 8.55 %. ^1^H NMR (400 MHz, C_6_D_6_): *δ*=7.21–7.11 (m, 10 H), 7.01 (m, *J=*7.4 Hz, 2 H), 6.96–6.86 (m, 1 H), 6.49 (t, *J=*7.1 Hz, 1 H), 6.40 (m, *J=*7.9 Hz, 2 H), 4.95 (s, 1 H), 4.10 (s, 2 H), 3.26 (hept, *J=*6.8 Hz, 4 H), 2.96–2.79 (m, 4 H), 1.77 (s, 6 H), 1.22 (d, *J=*6.8 Hz, 12 H), 1.17 (d, *J=*6.9 Hz, 12 H), 0.99–0.92 (m, 4 H) ppm. ^13^C{^1^H} NMR (101 MHz, C_6_D_6_): *δ*=165.8, 159.6, 146.0, 145.6, 141.8, 130.1, 128.5, 128.2, 127.0, 125.4, 124.4, 123.8, 111.7, 110.3, 93.8, 68.4, 52.1, 28.2, 24.7, 24.3, 24.3, 24.2 ppm.

CCDC https://www.ccdc.cam.ac.uk/services/structures?id=doi:10.1002/chem.201904148 contains the supplementary crystallographic data for this paper. These data are provided free of charge by http://www.ccdc.cam.ac.uk/.

## Conflict of interest

The authors declare no conflict of interest.

## Supporting information

As a service to our authors and readers, this journal provides supporting information supplied by the authors. Such materials are peer reviewed and may be re‐organized for online delivery, but are not copy‐edited or typeset. Technical support issues arising from supporting information (other than missing files) should be addressed to the authors.

Supplementary
