# Supplementary material for: Early Main Group Metal Catalysts for Imine Hydrosilylation
Source: Chemistry. 2019 Nov 18;25(70):16141–7. doi: 10.1002/chem.201904148 (PMC6973166; doi:10.1002/chem.201904148)
Supplement: Supplementary file 1 — Supplementary [file CHEM-25-16141-s001.pdf]

CHEMISTRY
A European Journal

Supporting Information

Early Main Group Metal Catalysts for Imine Hydrosilylation

Holger Elsen, Christian Fischer, Christian Knüpfer, Ana Escalona, and Sjoerd Harder*[a]

chem_201904148_sm_miscellaneous_information.pdf

Supporting Information

Table of Contents

Selected NMR spectra	S2
Details for investigations on reaction intermediates	S3
GCMS analysis	S7
Crystal structure determination	S8
Calculational details	S10
References	S27

Selected NMR spectra

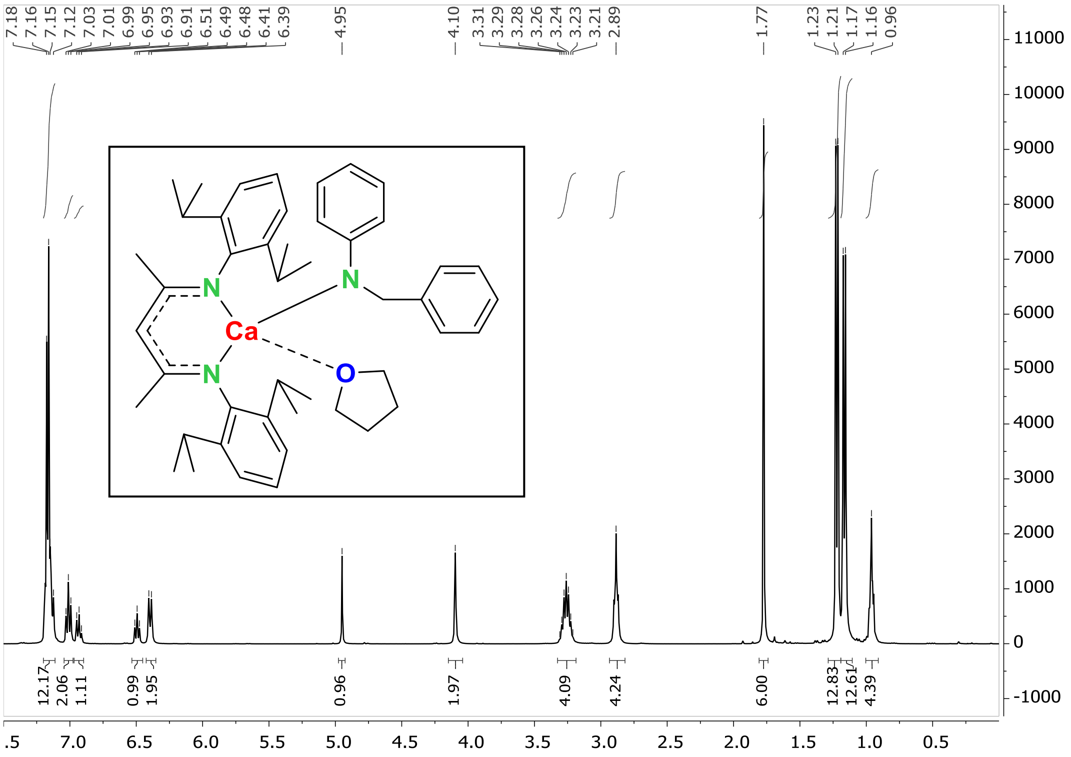

Figure S1: ^{1}H NMR spectrum of (^{DIPP}BDI)Ca[N(Ph)CH$_2$Ph]·(THF) in C_6D_6.

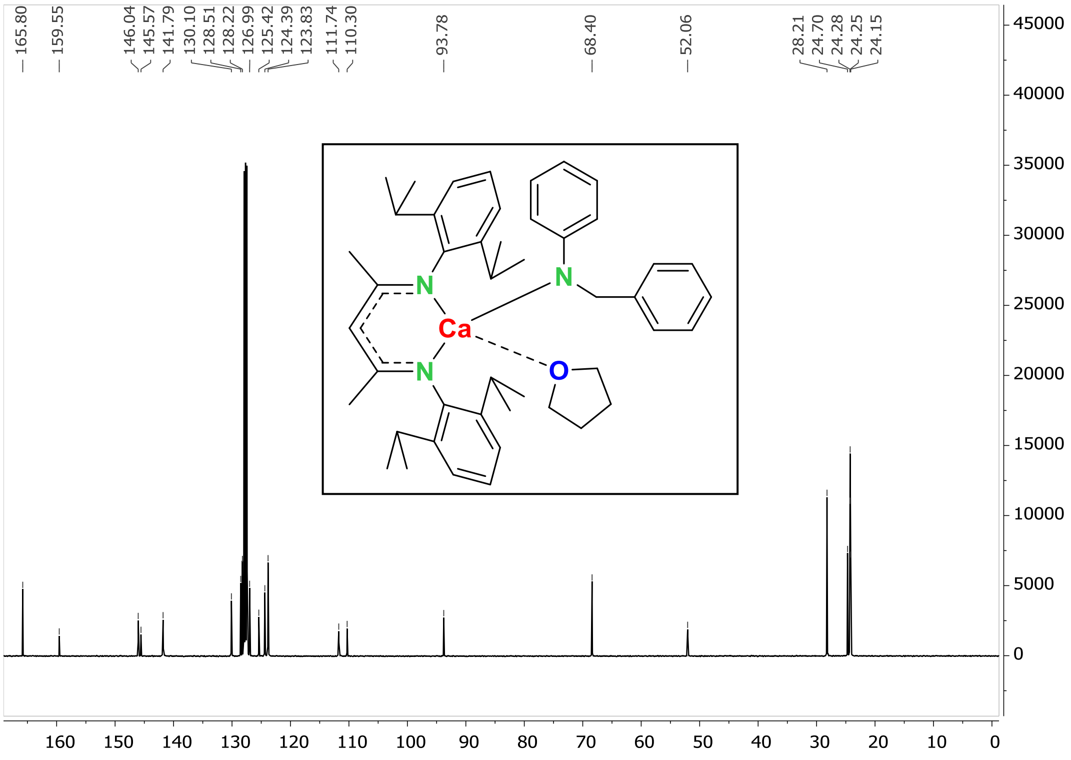

Figure S2: ^{13}C NMR spectrum of (^{DIPP}BDI)Ca[N(Ph)CH$_2$Ph]·(THF) in C_6D_6.

Details for investigations on reaction intermediates

To further investigate the proposed catalytic cycle, $[(BDI)CaH\cdot(THF)]_2$ (78.0mg, 0.0738mmol) was placed in a J-Young NMR tube and dissolved in 500 µl of C_6D_6. One equivalent of PhCH=N*t*Bu imine (23.8 mg, 26.2 µl, 0.147 mmol) was added and reduced to the corresponding amide at 60°C after 16 hours. After complete reduction (absence of a hydride peak and only trace amounts of imine), a slight excess of $PhSiH_3$ (17.6mg, 20.0 µl, 0.162 mmol) was added, giving the hydrosilylated amine after 20 hours at 60°C. Two further equivalents of imine and $PhSiH_3$ were added, which were hydrosilylated, showing that the reaction is indeed catalytic at 60°C. After the reaction, all volatiles were removed under reduced pressure to give a yellow solid. After washing with cold (-20°C) pentane (2 x 1ml) a white powder was received, which was identified as the catalyst $[(BDI)CaH\cdot(THF)]_2$ (16.1 mg, 0.0152 mmol, 21 %).

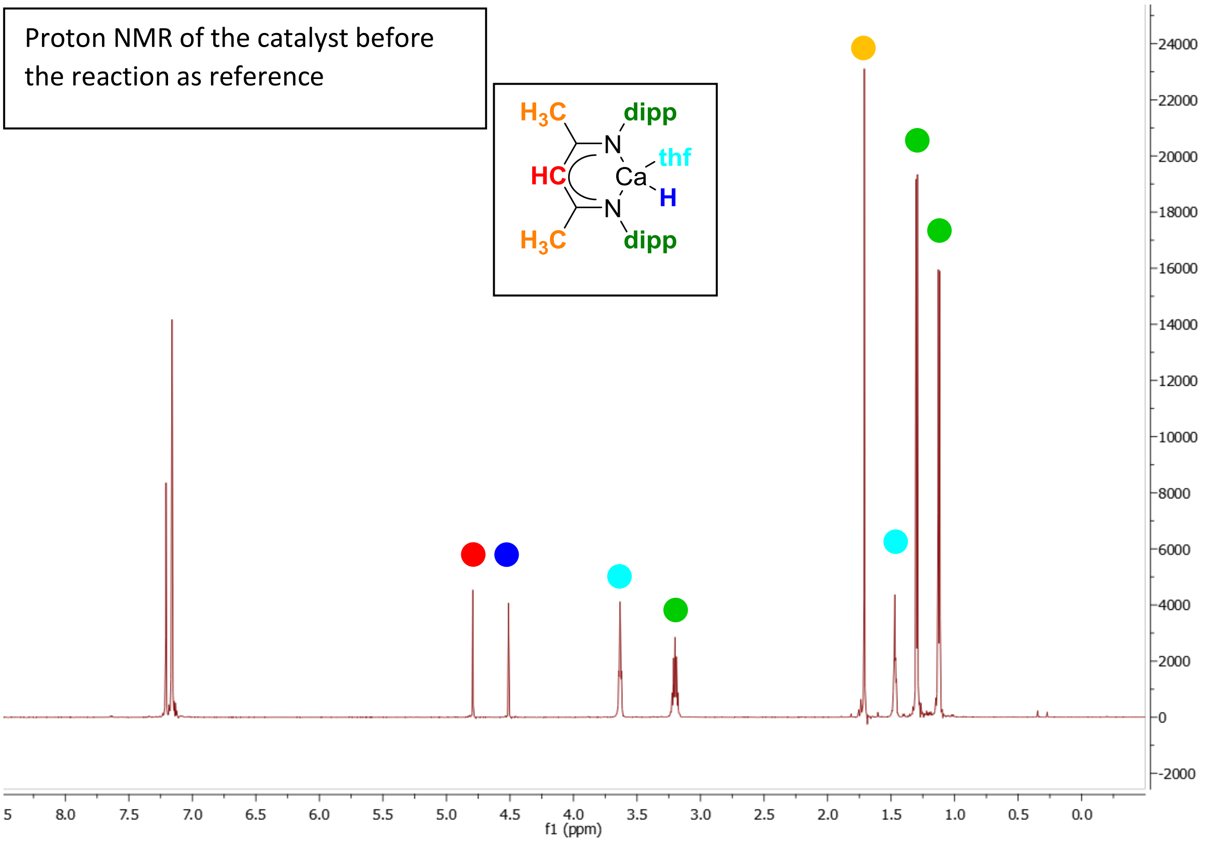

Figure S3: ^{1}H NMR spectrum of $[(^{DIPP}BDI)CaH\cdot(THF)]_2$ in C_6D_6.

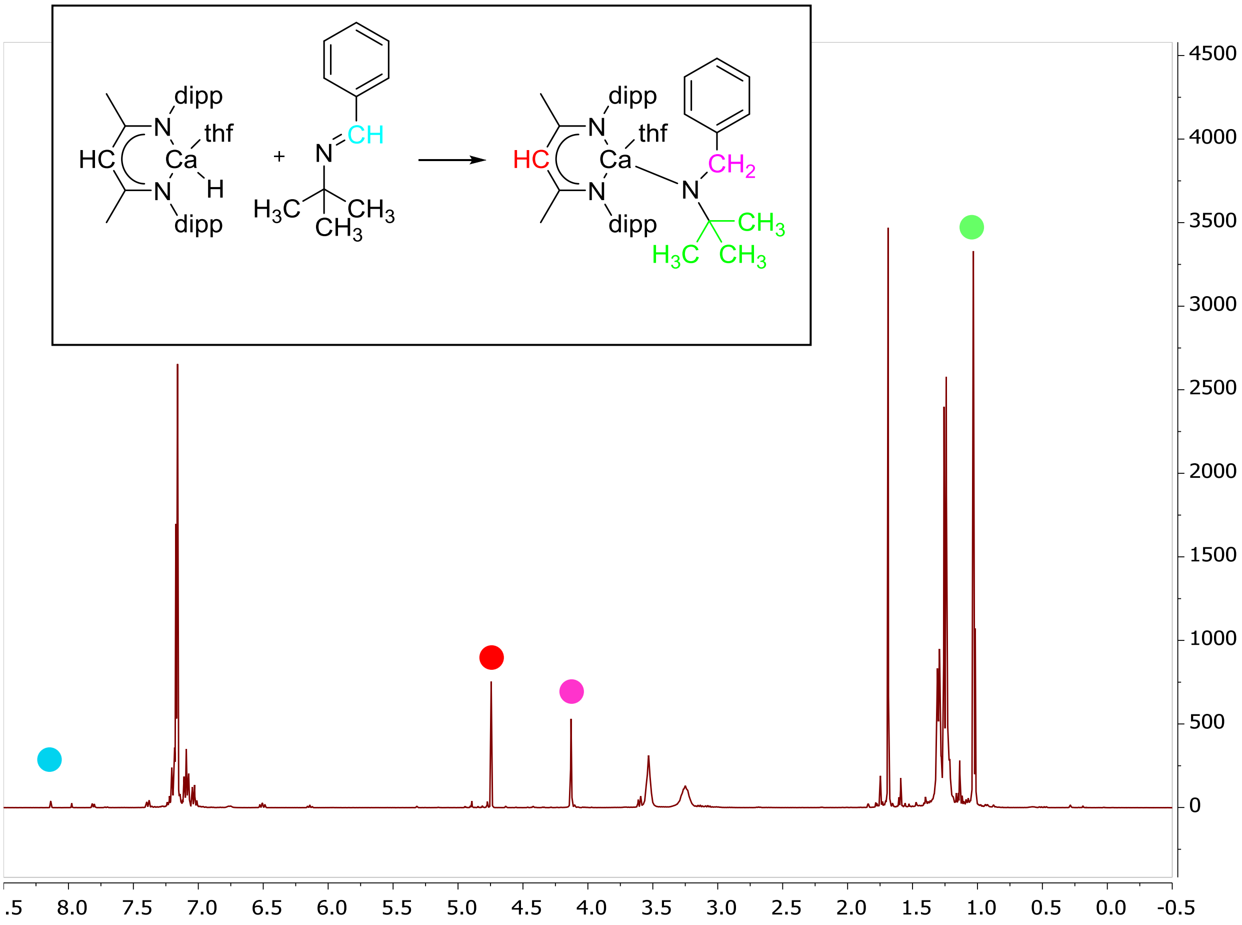

Figure S4: ^{1}H NMR of [(^{DIPP}BDI)CaH·(THF)]$_2$ in C_6D_6 after reaction with two equivalents of imine **I**.

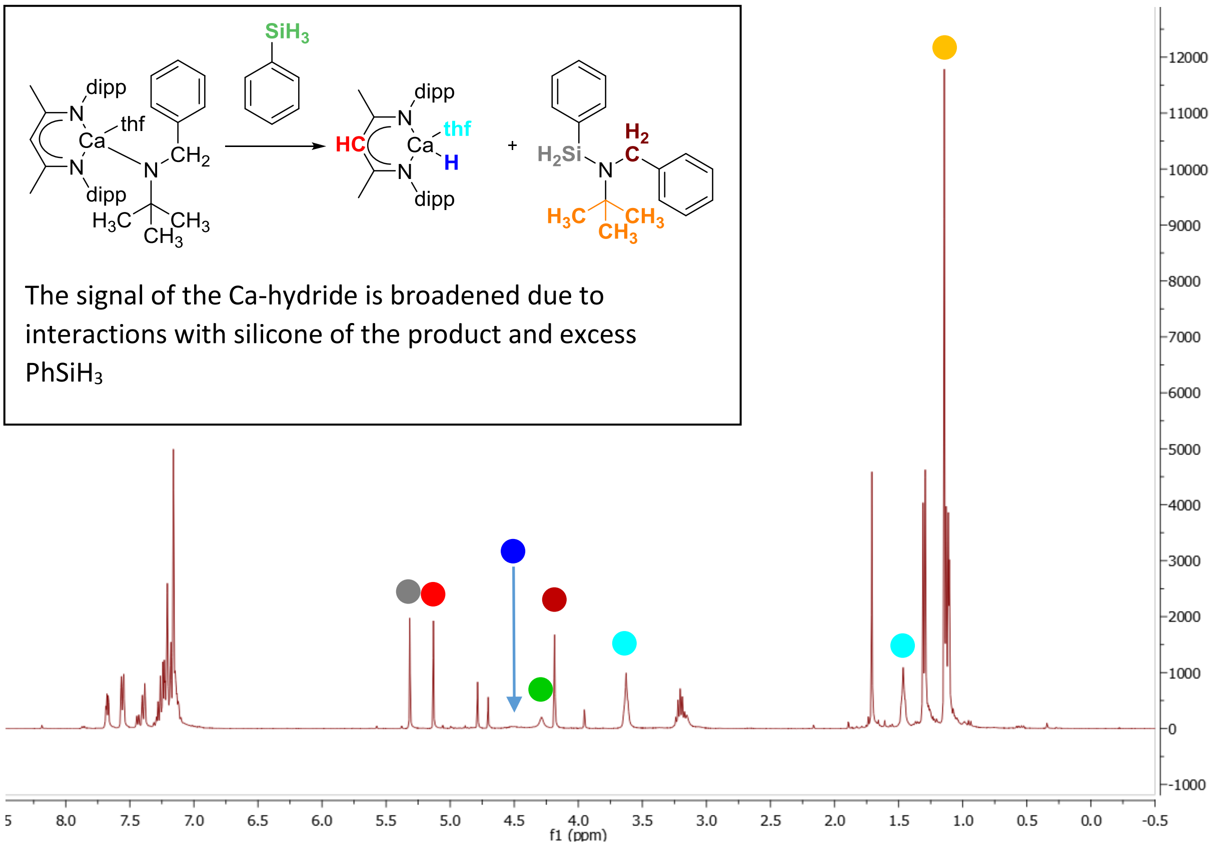

Figure S5: ^{1}H NMR of [(^{DIPP}BDI)CaH·(THF)]$_2$ in C_6D_6 after reaction with one equivalent of imine **I** and an slight excess of $PhSiH_3$.

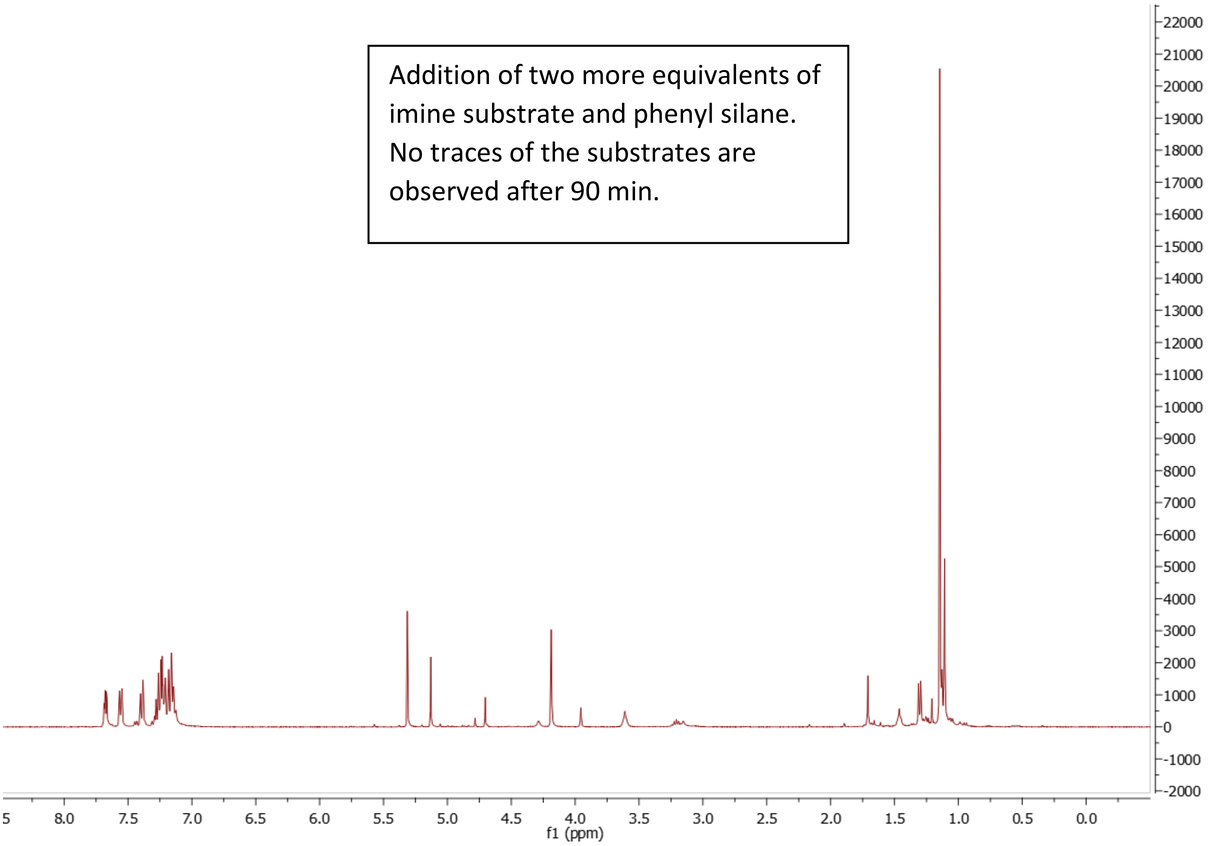

Figure S6: ^{1}H NMR of reaction mixture after addition of two more equivalents of imine **I** and $PhSiH_3$.

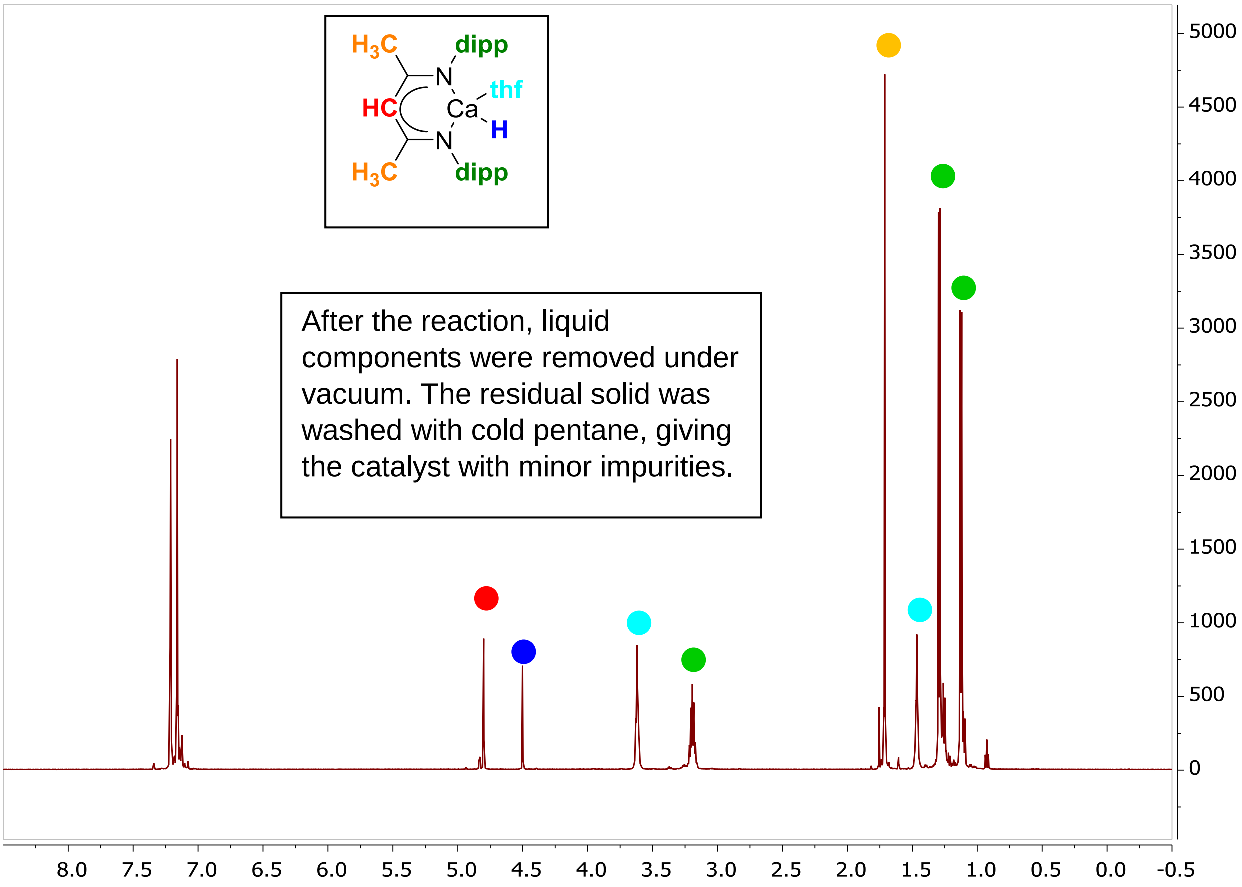

Figure S7: ^{1}H NMR spectrum of the recycled catalyst [(^{DIPP}BDI)CaH·(THF)]$_2$ in C_6D_6.

GC-MS analysis

GC/MS measurements were performed on a Thermo Scientific™ Trace™ 1310 gas chromatography system (carrier gas Helium) with detection by a Thermo Scientific™ ISQ™ LT Single Quadrupole mass spectrometer.

A Thermo Scientific™ TraceGOLD™ TG-5SilMS GC Column of the dimensions 0.25 mm x 30 m with a film thickness of 0.25 µm was used. The samples (1 µL) were injected with an Instant Connect-SSL Module in the split mode (Injector Temperature: 350 °C). Temperature programs were started at 40 °C followed by heating ramps, optimized for every separation problem, until 330 °C. Baseline separation of each analyte was achieved by choosing different temperature programs.

Peaks in the chromatogram were identified by comparing the obtained EI-MS-spectra with the entrances in NIST/EPA/NIH mass spectral library (version 2.2, built June 10 2014). All assigned peaks refer to entrances having a match and reverse match greater than 850. Furthermore samples, containing substrates and/or catalysis products were prepared for peak identification and calibration.

Crystal structure determination

Crystal structure of (^{DIPP}BDI)Ca[N(Ph)CH_2Ph]·(THF)·$(C_6H_6)_{0.5}$ (13). Using the program Olex2,[S8] the structure was solved by Direct Methods (ShelXT)[S9] and refined by Least Squares minimisation with ShelXL.[S10] The hydrogen atoms have been placed on calculated positions and were refined isotropically in a riding model. One of the *iso*propyl-groups was refined over two positions (ratio ~ 58:42) and the respective parts were idealized by distance restraints (SADI). Half a benzene molecule cocrystallized in the asymmetric unit.

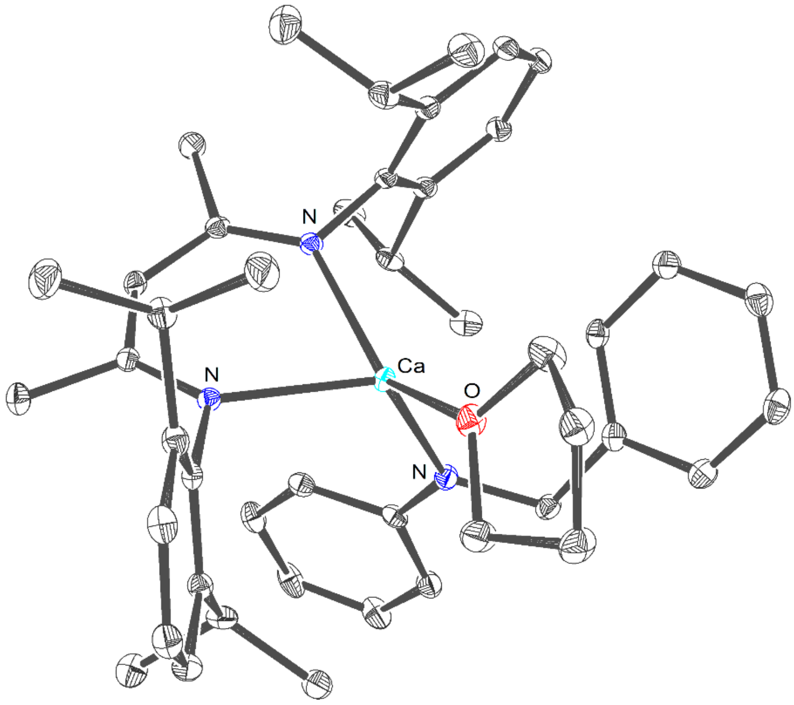

Figure S8. ORTEP plot (30% probability) for (^{DIPP}BDI)Ca[N(Ph)CH_2Ph]·(THF).

**Table S1.** Crystal data and structure refinement for (^{DIPP}BDI)Ca[N(Ph)CH_2Ph]·(THF)·$(C_6H_6)_{0.5}$	
Identification code	hasj171116a
Empirical formula	$C_{49}H_{64}CaN_3O$
Formula weight	751.11
Temperature/K	99.98(10)
Crystal system	monoclinic
Space group	$P2_1/n$
a/Å	12.10680(10)
b/Å	17.18280(10)
c/Å	20.5840(2)
α/°	90
β/°	93.1520(10)
γ/°	90
Volume/Å^3	4275.58(6)
Z	4
ρ_{calc}g/cm^3	1.167
μ/mm^{-1}	1.550
F(000)	1628.0
Crystal size/mm^3	0.243 × 0.166 × 0.091
Color crystal	colorless
Radiation	CuKα (λ = 1.54184)
2Θ range for data collection/°	6.706 to 147.318
Index ranges	-12 ≤ h ≤ 14, -21 ≤ k ≤ 21, -25 ≤ l ≤ 25
Reflections collected	33301
Independent reflections	8465 [R_{int} = 0.0241, R_{sigma} = 0.0199]
Data/restraints/parameters	8465/7/527
Goodness-of-fit on F^2	1.035
Final R indexes [I>=2σ (I)]	R_1 = 0.0324, wR_2 = 0.0817
Final R indexes [all data]	R_1 = 0.0343, wR_2 = 0.0833
Largest diff. peak/hole / e Å^{-3}	0.27/-0.32

Calculational details

All calculations were carried out using Gaussian 09 Rev. D.[S11] All methods were used as implemented. All structures were fully optimized on a B3PW91/6-311++G**[S12-S15] level of theory. Structures were determined to be true minima (NIMAG=0), except the two transition states (NIMAG=1). Solvent effects were modeled via PCM, using benzene as solvent.[S16] Structures were drawn and evaluated using Molecule V2.3.[S17]

Coordinates

	3		
CaH2			
Ca	0.000000	0.000000	0.051982
H	0.000000	-1.946219	-0.519825
H	0.000000	1.946219	-0.519825

	27		
Imine I (A)			
N	-1.117185	-0.359324	0.000026
C	-0.225927	0.543097	-0.000021
C	-2.554836	-0.047210	-0.000006
C	-2.917489	1.446839	-0.000689
C	-3.134120	-0.722396	-1.257607
C	-3.133991	-0.721258	1.258333
H	-4.003905	1.563889	-0.000735
H	-2.535123	1.962542	0.884986
H	-2.535125	1.961724	-0.886849
H	-4.223282	-0.624950	-1.285127
H	-2.727302	-0.267289	-2.164732
H	-2.878685	-1.783765	-1.270569
H	-4.223136	-0.623709	1.285896
H	-2.878599	-1.782625	1.272209
H	-2.727012	-0.265322	2.164964
H	-0.460802	1.615784	-0.000107
C	1.217274	0.230127	-0.000022
C	1.678960	-1.093932	-0.000048
C	3.041196	-1.361247	-0.000026
C	3.964938	-0.312869	0.000027
C	2.149746	1.273722	0.000005
C	3.516671	1.005435	0.000039
H	0.948833	-1.894845	-0.000091
H	3.389573	-2.389344	-0.000046
H	5.029331	-0.525617	0.000043
H	1.801306	2.303250	-0.000014
H	4.229694	1.823697	0.000081

	30		
A1			
Ca	0.358479	2.156219	-0.443932
N	1.032677	-0.207698	0.137785
H	-0.078550	3.290534	1.231263
H	0.734431	2.393441	-2.460902
C	0.118795	-1.084395	-0.010233
C	2.467941	-0.581476	0.121605
C	2.779032	-1.789059	-0.766926
C	3.225493	0.647320	-0.393761
C	2.866952	-0.865138	1.576568

H	3.860477	-1.941745	-0.803327
H	2.341127	-2.715902	-0.386044
H	2.427227	-1.626458	-1.789812
H	4.298040	0.446080	-0.450136
H	2.885476	0.939027	-1.394544
H	3.100181	1.494983	0.291771
H	3.943872	-1.043998	1.642142
H	2.619678	-0.017470	2.220869
H	2.346664	-1.748368	1.958168
H	0.349122	-2.130805	-0.235462
C	-1.308454	-0.748472	0.079914
C	-1.761100	0.335544	0.847674
C	-3.112260	0.661397	0.866607
C	-4.024659	-0.097597	0.137833
C	-2.237587	-1.521744	-0.626391
C	-3.586519	-1.192565	-0.603280
H	-1.071546	0.907818	1.467468
H	-3.447848	1.502869	1.463006
H	-5.079123	0.156790	0.157201
H	-1.895803	-2.371431	-1.210106
H	-4.297558	-1.788746	-1.165065

	30		
A2*			
C	-0.167651	-0.770559	-0.424382
N	-1.047269	-0.234082	0.365756
Ca	-0.233610	1.908617	-0.457448
H	-0.398045	3.902739	-0.028193
C	-2.485891	-0.510272	0.193549
C	1.276718	-0.576666	-0.126957
H	-0.400076	-1.556127	-1.141835
H	-0.052959	0.501469	-2.031639
C	-3.068199	-0.664676	1.601957
C	-3.103404	0.722421	-0.489642
C	-2.795701	-1.759869	-0.635504
C	1.682928	0.033085	1.066539
C	3.038325	0.225475	1.336971
C	3.996980	-0.215883	0.434617
C	2.254036	-1.025442	-1.025006
C	3.599180	-0.853425	-0.743348
H	0.930868	0.304432	1.802381
H	3.339710	0.700898	2.264555
H	5.051519	-0.074448	0.646705
H	1.941737	-1.482975	-1.958280
H	4.346372	-1.204298	-1.447567
H	-4.178911	0.594621	-0.638147
H	-2.980149	1.624134	0.125137
H	-2.662864	0.880389	-1.483285
H	-3.874098	-1.939859	-0.638700
H	-2.477907	-1.653492	-1.676562
H	-2.312547	-2.645207	-0.210522
H	-4.155489	-0.783169	1.563540
H	-2.642214	-1.541238	2.097829
H	-2.835802	0.213243	2.210486

	30		
A3			
Ca	0.430464	1.605863	-0.000102

N	-1.054211	-0.071417	-0.000016
H	0.595372	3.648645	0.000260
C	-0.391244	-1.362872	0.000069
C	-2.515497	-0.111518	0.000002
C	-3.079879	-0.805887	1.254672
C	-2.987147	1.346933	-0.000106
C	-3.079905	-0.806085	-1.254547
H	-4.173869	-0.755828	1.277226
H	-2.803399	-1.864142	1.289963
H	-2.695354	-0.323533	2.158846
H	-4.078884	1.420809	-0.000087
H	-2.621784	1.873455	0.889717
H	-2.621828	1.873306	-0.890036
H	-4.173895	-0.756008	-1.277099
H	-2.695380	-0.323888	-2.158806
H	-2.803447	-1.864351	-1.289666
H	-0.620674	-1.986376	0.883924
C	1.085184	-1.051912	0.000057
C	1.751516	-0.783181	-1.203068
C	3.042423	-0.249281	-1.205356
C	3.685295	0.029967	-0.000002
C	1.751494	-0.782998	1.203152
C	3.042401	-0.249096	1.205382
H	1.242611	-0.988232	-2.140986
H	3.543830	-0.047966	-2.146530
H	4.683368	0.454378	-0.000025
H	1.242570	-0.987899	2.141092
H	3.543788	-0.047631	2.146535
H	-0.620666	-1.986488	-0.883708

	45		
A4			
Ca	0.227404	-0.153703	-0.338991
N	-1.946411	-0.588806	-0.084448
H	1.309316	-0.646789	-2.049033
C	-2.776302	0.593401	-0.005166
C	-2.661969	-1.860890	-0.094414
C	-3.555582	-2.019860	-1.341208
C	-1.592969	-2.960374	-0.131598
C	-3.521351	-2.064445	1.168954
H	-4.036968	-3.003891	-1.364751
H	-4.351828	-1.269628	-1.363905
H	-2.955548	-1.905772	-2.249032
H	-2.036812	-3.960329	-0.162652
H	-0.960634	-2.854458	-1.022386
H	-0.961243	-2.906757	0.763757
H	-3.978090	-3.060245	1.184769
H	-2.904419	-1.953366	2.066792
H	-4.334224	-1.333878	1.225801
H	-3.526891	0.661706	-0.815756
C	-1.863364	1.792332	-0.117939
C	-1.371344	2.441689	1.019406
C	-0.451208	3.485413	0.908492
C	0.011611	3.879355	-0.343984
C	-1.385341	2.197642	-1.372107
C	-0.454107	3.229855	-1.486069
H	-1.731854	2.133174	1.996727
H	-0.099803	3.993228	1.801538

H	0.726371	4.691089	-0.432292
H	-1.755749	1.697547	-2.262834
H	-0.100335	3.531040	-2.466695
H	-3.345752	0.684042	0.939500
H	3.225602	-2.440454	-2.267476
H	5.242039	-1.514514	-1.383982
Si	3.798332	-1.205109	-1.686071
H	3.812442	-0.028304	-2.585144
C	3.033817	-0.794644	-0.002684
C	3.010668	0.523569	0.487029
C	2.426769	-1.787593	0.787784
C	2.347087	0.847946	1.670036
C	1.763383	-1.470391	1.972723
C	1.700085	-0.145029	2.409017
H	1.172713	0.105939	3.322688
H	1.286217	-2.253871	2.552434
H	2.437039	-2.820455	0.451430
H	2.325658	1.877524	2.011915
H	3.483584	1.316792	-0.085094

	45		
A5*			
Ca	1.578343	0.716732	1.610900
N	0.800937	-1.057120	-0.079141
H	3.453391	0.538804	2.452331
C	0.558889	-0.200923	-1.282711
C	1.883779	-2.103540	-0.283313
C	3.230640	-1.426704	-0.576161
C	2.046142	-2.898049	1.018292
C	1.557909	-3.074234	-1.429757
H	4.007419	-2.192719	-0.653229
H	3.228501	-0.886557	-1.526103
H	3.539745	-0.748503	0.230078
H	2.870927	-3.606625	0.906487
H	2.293204	-2.247654	1.864130
H	1.153902	-3.476817	1.268964
H	2.369191	-3.800124	-1.538839
H	0.635140	-3.624943	-1.240317
H	1.457928	-2.558429	-2.389228
H	1.219758	-0.517052	-2.090959
C	0.794719	1.272402	-1.001275
C	-0.235876	2.067829	-0.484285
C	0.003425	3.388826	-0.108879
C	1.277004	3.936690	-0.235236
C	2.066170	1.845732	-1.148398
C	2.309130	3.163052	-0.762505
H	-1.225579	1.643398	-0.360336
H	-0.808042	3.984292	0.295708
H	1.464916	4.960482	0.070305
H	2.879518	1.258056	-1.559995
H	3.303360	3.581808	-0.875142
H	-0.462444	-0.325192	-1.655385
H	-0.982528	-2.983985	0.002564
H	-0.531630	-2.012146	2.123745
Si	-0.735396	-1.676382	0.701233
H	-0.359650	0.446655	2.250488
C	-2.327937	-0.751357	0.292096
C	-3.019242	-1.115848	-0.874041

C	-2.931136	0.178258	1.150876
C	-4.248836	-0.545540	-1.193887
C	-4.172063	0.729521	0.846998
C	-4.828315	0.378940	-0.330668
H	-5.791664	0.818388	-0.570817
H	-4.628098	1.438839	1.531177
H	-2.392134	0.476213	2.046017
H	-4.759016	-0.833869	-2.107742
H	-2.600395	-1.868105	-1.538064

45
A6

N	-0.117362	0.462275	-0.097234
C	-0.693653	-0.387653	-1.186763
C	0.292279	1.852485	-0.540931
C	-0.912826	2.622399	-1.096818
C	0.803930	2.621137	0.681315
C	1.397167	1.809643	-1.607312
H	-0.600444	3.641250	-1.342677
H	-1.296539	2.183683	-2.021606
H	-1.727535	2.707796	-0.365076
H	1.058855	3.641262	0.383597
H	0.043694	2.693679	1.465660
H	1.706525	2.175419	1.107304
H	1.655028	2.828108	-1.912538
H	2.302099	1.328664	-1.231657
H	1.077519	1.276286	-2.507583
H	-0.672873	0.165671	-2.126287
C	-2.118135	-0.812746	-0.887156
C	-3.205740	-0.018641	-1.278430
C	-4.504383	-0.338551	-0.886853
C	-4.740400	-1.465470	-0.102039
C	-2.374615	-1.951164	-0.112619
C	-3.673125	-2.274509	0.276968
H	-3.039554	0.865993	-1.882625
H	-5.328049	0.298523	-1.190017
H	-5.749223	-1.708928	0.213245
H	-1.551618	-2.578962	0.208896
H	-3.843371	-3.150341	0.893058
H	-0.084835	-1.282873	-1.351702
H	1.021768	0.217379	2.317630
Si	0.980979	-0.453981	1.000430
H	0.360795	-1.796718	1.072262
C	2.760965	-0.671021	0.415438
C	3.801542	0.012350	1.060028
C	3.101271	-1.551517	-0.622886
C	5.127493	-0.158078	0.671684
C	4.423520	-1.723280	-1.017775
C	5.439496	-1.022739	-0.372294
H	6.472302	-1.158565	-0.676927
H	4.664326	-2.409681	-1.823517
H	2.327472	-2.122729	-1.129258
H	5.916791	0.379744	1.187401
H	3.576838	0.682833	1.885553
Ca	-2.298928	0.653606	1.528484
H	-1.690451	-0.651322	3.001335
H	-3.081835	2.565648	1.569137

	15		
PhSiH3			
H	-2.877775	-0.756174	-1.167108
H	-2.828643	1.406705	-0.065750
Si	-2.343477	0.000275	0.000754
H	-2.878781	-0.638340	1.236809
C	-0.464961	-0.001312	-0.001663
C	0.254607	1.200219	-0.001322
C	0.257980	-1.202757	-0.001251
C	1.646987	1.203646	0.000456
C	1.648263	-1.203586	0.000437
C	2.345836	0.001533	0.001372
H	3.431149	0.002061	0.002312
H	2.188825	-2.144821	0.000481
H	-0.267557	-2.154345	-0.002925
H	2.185098	2.146285	0.000525
H	-0.275910	2.148316	-0.003076

	42		
A7			
N	0.275340	0.306914	-0.011788
C	1.232691	-0.191187	0.969072
C	0.138409	1.783448	-0.122829
C	1.511332	2.457179	-0.255966
C	-0.675400	2.122157	-1.375770
C	-0.594026	2.347362	1.107284
H	1.380080	3.538804	-0.358116
H	2.144841	2.284757	0.617290
H	2.044915	2.089617	-1.135359
H	-0.786426	3.206647	-1.459778
H	-0.179441	1.760592	-2.280267
H	-1.684710	1.699191	-1.335002
H	-0.685699	3.436085	1.037528
H	-1.598057	1.922438	1.184501
H	-0.056385	2.125399	2.033843
H	1.285812	0.490427	1.826517
C	2.647212	-0.454631	0.474105
C	3.701740	-0.469234	1.390181
C	4.998203	-0.767642	0.984537
C	5.261379	-1.057068	-0.351663
C	2.921990	-0.744690	-0.860809
C	4.218137	-1.044085	-1.271968
H	3.506176	-0.240901	2.435403
H	5.804856	-0.769194	1.711071
H	6.272005	-1.287871	-0.672612
H	2.113510	-0.722641	-1.584086
H	4.413376	-1.266746	-2.316519
H	0.858632	-1.135751	1.383138
H	-0.847663	-0.744192	-2.230306
Si	-0.825646	-0.831325	-0.740650
H	-0.279660	-2.160216	-0.348103
C	-2.633732	-0.728795	-0.207546
C	-3.652529	-0.682926	-1.167821
C	-3.006437	-0.745458	1.144256
C	-4.994462	-0.654063	-0.796272
C	-4.344590	-0.715972	1.522173
C	-5.341753	-0.669410	0.550578
H	-6.386540	-0.646260	0.844154

H	-4.612212	-0.731079	2.574276
H	-2.241846	-0.781915	1.916129
H	-5.767950	-0.617984	-1.557232
H	-3.394940	-0.668962	-2.224099

25

Imine II (B)

C	0.394825	0.385377	-0.177377
N	-0.456175	-0.515563	0.122243
C	-1.825473	-0.225515	0.070538
C	1.841124	0.172761	-0.086600
H	0.080321	1.375536	-0.539038
C	2.376426	-1.037380	0.374525
C	3.749978	-1.207770	0.449106
C	4.608812	-0.175618	0.067786
C	2.708812	1.201697	-0.467289
C	4.086465	1.029326	-0.390879
H	1.694294	-1.827842	0.667191
H	4.159182	-2.147079	0.806914
H	5.683554	-0.313398	0.128849
H	2.298147	2.141656	-0.825757
H	4.751195	1.833589	-0.688420
C	-2.366721	0.978928	0.540592
C	-3.738315	1.199773	0.484968
C	-4.587487	0.229656	-0.038934
C	-2.689502	-1.211474	-0.421921
C	-4.055658	-0.976392	-0.491640
H	-1.712596	1.724342	0.981501
H	-4.146335	2.131878	0.863195
H	-5.657327	0.404791	-0.079671
H	-2.264893	-2.151646	-0.756915
H	-4.712040	-1.743097	-0.890805

28

B1

C	-0.434530	-0.958832	-0.170217
N	0.434361	-0.016716	-0.145578
Ca	0.137706	2.357088	0.654146
H	-0.662829	3.382689	-0.951340
C	1.815460	-0.331117	-0.139190
C	-1.874849	-0.725307	-0.183953
H	-0.109840	-2.005874	-0.186376
H	0.494134	2.638587	2.658049
C	-2.426941	0.499956	-0.589607
C	-3.801611	0.683782	-0.555656
C	-4.638771	-0.346530	-0.129576
C	-2.725495	-1.765597	0.212705
C	-4.100113	-1.573028	0.249656
H	-1.798134	1.303241	-0.978209
H	-4.219281	1.631976	-0.876663
H	-5.713182	-0.196825	-0.106579
H	-2.303246	-2.721949	0.507411
H	-4.751273	-2.378773	0.570868
C	2.363070	-1.288979	0.719319
C	3.732845	-1.523350	0.708622
C	4.563691	-0.808482	-0.149409
C	2.653238	0.404353	-0.984244
C	4.020424	0.154976	-0.994512

H	1.722385	-1.816247	1.418378
H	4.154481	-2.258162	1.386313
H	5.632535	-0.992138	-0.149468
H	2.218990	1.140275	-1.655597
H	4.662119	0.717384	-1.664299

	28		
B2*			
C	0.333423	-0.887973	0.110977
N	-0.510456	0.002030	0.504771
Ca	0.257150	1.823256	-0.999747
H	0.869729	3.744201	-0.624002
C	-1.894639	-0.209014	0.334618
C	1.776517	-0.671544	0.213402
H	0.005000	-1.853906	-0.28375
H	0.269608	0.351489	-2.469769
C	2.306333	0.391722	0.962992
C	3.679835	0.594653	1.013847
C	4.538013	-0.266880	0.335528
C	2.651029	-1.540830	-0.452555
C	4.021187	-1.338524	-0.391318
H	1.640442	1.030339	1.536482
H	4.079781	1.420841	1.591356
H	5.610254	-0.107453	0.378193
H	2.244486	-2.357709	-1.039951
H	4.690183	-2.009844	-0.918804
C	-2.423213	-0.845119	-0.797252
C	-3.801172	-0.986767	-0.926433
C	-4.656442	-0.502179	0.058052
C	-2.759188	0.312186	1.305059
C	-4.131099	0.150039	1.172516
H	-1.755738	-1.170922	-1.588564
H	-4.206607	-1.468971	-1.80993
H	-5.729837	-0.617301	-0.048631
H	-2.336339	0.821680	2.164572
H	-4.794291	0.539602	1.937743

	28		
C3			
C	-0.470837	-1.195305	-1.614656
N	0.419152	-0.054281	-1.463008
Ca	-0.410721	1.510669	0.068431
H	-1.008493	3.444381	0.209998
C	1.553518	-0.170547	-0.725014
C	-1.592600	-0.941632	-0.627940
H	-0.868150	-1.215462	-2.635550
H	-0.005209	-2.171251	-1.435285
C	-2.664246	-0.109604	-0.988718
C	-3.551241	0.382707	-0.030246
C	-3.389010	0.048953	1.312748
C	-1.453421	-1.284526	0.728772
C	-2.342303	-0.794831	1.685947
H	-2.789828	0.166722	-2.031961
H	-4.362846	1.035622	-0.332913
H	-4.071565	0.438938	2.059415
H	-0.637518	-1.932037	1.033809
H	-2.218199	-1.076048	2.727061
C	2.086507	-1.342206	-0.110833

C	3.210831	-1.285517	0.695993
C	3.868186	-0.081746	0.966665
C	2.259897	1.044552	-0.446827
C	3.384192	1.077950	0.374614
H	1.627890	-2.307373	-0.293846
H	3.587270	-2.207811	1.130466
H	4.744340	-0.058869	1.604139
H	1.991061	1.936311	-1.015533
H	3.894754	2.023986	0.529592

43

B4

Ca	-0.315453	0.319978	-0.743511
N	-2.482549	0.253982	-0.044153
H	0.924808	-0.620756	-2.069567
C	-2.858886	1.524096	0.551289
C	-3.451424	-0.722627	-0.060301
H	-3.719983	1.993233	0.044104
C	-1.650046	2.417842	0.417361
C	-0.638697	2.395428	1.386241
C	0.589383	3.020254	1.155365
C	0.824869	3.673797	-0.053026
C	-1.405703	3.094879	-0.785367
C	-0.179611	3.720733	-1.019310
H	-0.817324	1.873800	2.322391
H	1.362647	2.991008	1.916002
H	1.781843	4.149351	-0.238094
H	-2.183705	3.118278	-1.543428
H	-0.006850	4.239073	-1.956777
H	-3.132972	1.435854	1.617073
H	2.222040	-2.167758	2.418405
H	1.392608	-2.847699	0.229324
Si	2.092078	-1.781006	0.986494
H	1.165551	-0.587185	1.004714
C	3.776757	-1.326033	0.315705
C	4.879983	-1.297748	1.180474
C	3.976407	-1.030009	-1.041373
C	6.149421	-0.979402	0.706447
C	5.246354	-0.712705	-1.512147
C	6.332886	-0.686993	-0.641329
H	7.322763	-0.441916	-1.013596
H	5.387917	-0.487466	-2.564541
H	3.127974	-1.035944	-1.722378
H	6.993920	-0.964087	1.388139
H	4.751681	-1.530997	2.234061
C	-3.153614	-1.969149	-0.664043
C	-4.077631	-2.997192	-0.709677
C	-5.351519	-2.839954	-0.157394
C	-4.745802	-0.582471	0.488323
C	-5.668194	-1.624471	0.436586
H	-2.173738	-2.119600	-1.114741
H	-3.804694	-3.934859	-1.185357
H	-6.075452	-3.646481	-0.194812
H	-5.037784	0.347551	0.963244
H	-6.653024	-1.476407	0.871270

43

B5*

Ca	-0.872912	-1.077570	1.529957
N	-0.658910	0.554001	-0.284244
H	-1.566625	-1.374667	3.408518
C	-1.051116	-0.256115	-1.454089
C	-1.188884	1.861710	-0.202126
H	-1.637768	0.333187	-2.161469
C	-1.882010	-1.449599	-1.013616
C	-1.376030	-2.753519	-1.059773
C	-2.101684	-3.821749	-0.528032
C	-3.341347	-3.600393	0.062991
C	-3.141155	-1.243387	-0.427130
C	-3.862562	-2.307495	0.107280
H	-0.403583	-2.929416	-1.509874
H	-1.691427	-4.825457	-0.571317
H	-3.898221	-4.426678	0.490732
H	-3.548084	-0.236780	-0.382605
H	-4.829852	-2.127435	0.563825
H	-0.173176	-0.621495	-1.998892
H	1.431943	1.848005	-0.175282
H	1.022156	0.432213	1.739536
Si	1.298598	0.401493	0.237057
H	0.923408	-1.135251	0.069644
C	3.154676	-0.033082	0.022585
C	4.085002	0.991235	-0.190533
C	3.637111	-1.344092	0.101353
C	5.447362	0.722175	-0.294861
C	4.996371	-1.625792	0.000515
C	5.905212	-0.588920	-0.197366
H	6.966068	-0.803556	-0.282458
H	5.349035	-2.650751	0.069295
H	2.933002	-2.162884	0.241335
H	6.151960	1.532575	-0.456690
H	3.733775	2.017611	-0.274700
C	-1.315768	2.449811	1.067136
C	-1.802984	3.741053	1.224343
C	-2.181520	4.491851	0.116129
C	-1.546635	2.644209	-1.311554
C	-2.043802	3.932917	-1.150092
H	-1.022681	1.890794	1.952204
H	-1.892260	4.157173	2.222793
H	-2.568136	5.497928	0.236406
H	-1.418873	2.262381	-2.318367
H	-2.311820	4.510102	-2.029840

	43		
B6			
Ca	1.057517	1.148348	1.857372
N	0.167936	-0.559132	0.104451
H	2.299131	0.481513	3.351128
C	0.005912	0.411776	-1.007663
C	1.142906	-1.603738	-0.085831
H	0.033598	-0.088036	-1.981114
C	1.048112	1.513106	-0.952050
C	0.652349	2.834375	-0.704634
C	1.600049	3.848438	-0.567036
C	2.957152	3.555281	-0.660943
C	2.418559	1.234326	-1.062466
C	3.364045	2.246100	-0.910016

H	-0.402570	3.061044	-0.595671
H	1.273379	4.864902	-0.373857
H	3.695047	4.341585	-0.542083
H	2.749272	0.220645	-1.261914
H	4.419885	2.010140	-0.989858
H	-0.982496	0.863375	-0.913592
H	-1.336593	-2.610503	0.752202
H	-1.087055	-0.894271	2.430783
Si	-1.311500	-1.138139	0.981346
H	-0.901840	1.807951	1.845560
C	-2.921987	-0.460962	0.310532
C	-3.647376	-1.264722	-0.583456
C	-3.467156	0.773635	0.693835
C	-4.872773	-0.844283	-1.091575
C	-4.696547	1.188448	0.191484
C	-5.398490	0.384225	-0.702838
H	-6.357012	0.712231	-1.093278
H	-5.108874	2.144030	0.500533
H	-2.899798	1.407182	1.372030
H	-5.419783	-1.478097	-1.782467
H	-3.257537	-2.235277	-0.879484
C	1.910615	-2.015712	1.007782
C	2.839846	-3.044064	0.874389
C	3.022263	-3.674302	-0.350131
C	1.322025	-2.252405	-1.313408
C	2.257484	-3.271997	-1.442313
H	1.812294	-1.526571	1.976978
H	3.428356	-3.338067	1.737072
H	3.750617	-4.471184	-0.455178
H	0.724684	-1.974226	-2.174484
H	2.380483	-3.762619	-2.402706

40

B7

N	1.018592	0.102124	0.065204
C	0.323530	0.732739	-1.063137
C	2.409355	-0.094597	-0.037187
H	1.077548	1.229014	-1.682261
C	-0.690441	1.771013	-0.631691
C	-1.995301	1.740051	-1.122129
C	-2.912230	2.727155	-0.768180
C	-2.532865	3.755314	0.087296
C	-0.319496	2.807439	0.229759
C	-1.232334	3.791905	0.586998
H	-2.298755	0.932191	-1.781644
H	-3.924584	2.687955	-1.157675
H	-3.245832	4.524527	0.366265
H	0.692504	2.834253	0.622241
H	-0.929594	4.592161	1.254949
H	-0.174836	-0.009047	-1.701624
H	0.976455	-1.551380	2.064340
H	-0.529359	0.339955	2.284075
Si	0.051238	-0.648648	1.333106
C	-1.375574	-1.621479	0.593813
C	-1.141091	-2.643819	-0.337455
C	-2.697010	-1.375916	0.986962
C	-2.190484	-3.393094	-0.858891
C	-3.750341	-2.126373	0.471072

C	-3.497905	-3.134858	-0.453559
H	-4.317756	-3.720596	-0.857451
H	-4.768128	-1.922209	0.788744
H	-2.907852	-0.585000	1.701396
H	-1.989890	-4.181462	-1.577759
H	-0.125230	-2.861420	-0.658030
C	3.217303	-0.050009	1.109564
C	4.588422	-0.252972	1.026104
C	5.199399	-0.481665	-0.203873
C	3.034027	-0.332904	-1.271086
C	4.410975	-0.513824	-1.348864
H	2.765251	0.167849	2.071561
H	5.186632	-0.210314	1.931031
H	6.272038	-0.628280	-0.268221
H	2.439787	-0.399686	-2.175668
H	4.866490	-0.696944	-2.317266

29

Imine VII ©

C	0.460871	0.393383	0.000033
N	-0.495132	-0.431368	-0.000293
C	-1.897925	0.004774	0.000001
C	1.917997	-0.020340	-0.000034
H	0.308498	1.484332	0.000337
C	-2.532624	-0.613474	-1.253208
C	-2.532297	-0.614195	1.253012
C	-2.126706	1.519897	0.000415
H	-3.609243	-0.421502	1.276208
H	-2.368548	-1.694405	1.264871
H	-2.088035	-0.193318	2.159955
H	-3.200167	1.728118	0.000108
H	-1.699612	1.997090	0.888177
H	-1.698998	1.997537	-0.886807
H	-3.609646	-0.421188	-1.275783
H	-2.088952	-0.191757	-2.160064
H	-2.368392	-1.693600	-1.265892
C	2.573806	0.582847	1.254466
C	2.074947	-1.539407	-0.001085
C	2.574250	0.584658	-1.253422
H	3.136383	-1.808064	-0.001148
H	1.602866	-1.983745	0.878201
H	1.603120	-1.982514	-0.881135
H	3.646530	0.364968	1.264705
H	2.454414	1.671209	1.285444
H	2.134582	0.167275	2.166302
H	3.646984	0.366816	-1.263605
H	2.135375	0.170398	-2.166026
H	2.454843	1.673059	-1.282864

32

C1

C	-0.690653	-0.889130	0.081571
N	0.387878	-0.228192	-0.032612
Ca	0.662726	2.277033	-0.004166
H	2.126506	2.763895	-1.377986
C	1.709576	-0.916133	0.023658
C	-2.089969	-0.334051	-0.022582
H	-0.668632	-1.969876	0.262075

H	-0.286924	3.110408	1.631907
C	2.264200	-0.900451	-1.407528
C	2.609749	-0.061766	0.929105
C	1.659184	-2.348123	0.559009
H	3.570156	-0.559013	1.085417
H	2.850307	0.902108	0.459727
H	2.154351	0.102046	1.911080
H	2.677623	-2.739042	0.618198
H	1.226742	-2.394140	1.563643
H	1.098023	-3.020749	-0.095967
H	3.274589	-1.319307	-1.420845
H	1.635401	-1.499011	-2.073156
H	2.315702	0.122354	-1.794627
C	-2.773543	-0.548727	1.341417
C	-2.130623	1.147115	-0.394656
C	-2.816205	-1.166255	-1.097023
H	-3.163389	1.461585	-0.567863
H	-1.756720	1.786427	0.421017
H	-1.588577	1.335107	-1.331329
H	-3.821344	-0.239563	1.284545
H	-2.752743	-1.602158	1.638037
H	-2.287202	0.041986	2.122148
H	-3.862694	-0.855271	-1.162641
H	-2.360419	-1.031946	-2.082608
H	-2.800724	-2.234028	-0.857008

	32		
C2*			
C	-0.518095	-0.559920	0.442863
N	0.466615	-0.232237	-0.325562
Ca	0.185228	2.144409	0.039077
H	0.716284	3.925947	-0.829364
C	1.851937	-0.654125	-0.004045
C	-1.971157	-0.483025	-0.013184
H	-0.375904	-1.192692	1.322302
H	-0.682053	1.275864	1.747873
C	2.425424	-1.231388	-1.303261
C	2.653002	0.598824	0.390081
C	1.967148	-1.689708	1.117300
H	3.699722	0.349962	0.580397
H	2.665148	1.348572	-0.413922
H	2.270324	1.040527	1.321248
H	3.013811	-1.983202	1.234110
H	1.625254	-1.297879	2.079489
H	1.395178	-2.593326	0.886364
H	3.479568	-1.498064	-1.178954
H	1.875054	-2.129133	-1.597229
H	2.343084	-0.504776	-2.115618
C	-2.911117	-0.538966	1.193006
C	-2.278978	0.751949	-0.862367
C	-2.171766	-1.748017	-0.878216
H	-3.263064	0.661080	-1.329567
H	-2.331136	1.651166	-0.232643
H	-1.557431	0.878313	-1.679125
H	-3.954258	-0.545796	0.862233
H	-2.743244	-1.448667	1.778790
H	-2.742201	0.321304	1.844523
H	-3.218620	-1.825313	-1.187906

H	-1.541864	-1.715362	-1.770832
H	-1.922914	-2.654788	-0.317841

	32		
C3			
C	0.715152	-0.762979	-0.597976
N	-0.435587	-0.031624	-0.111781
Ca	-0.715964	2.149463	0.057740
H	-0.854698	3.980653	-0.864233
C	-1.669812	-0.812485	-0.019318
C	2.067559	-0.295278	-0.005103
H	0.648929	-1.842133	-0.380709
H	0.812107	-0.700755	-1.700369
C	-2.803233	0.161179	0.350519
C	-2.051105	-1.484001	-1.354359
C	-1.608572	-1.884852	1.087990
H	-3.008840	-2.011884	-1.284335
H	-2.126066	-0.735055	-2.149716
H	-1.294763	-2.213874	-1.655132
H	-2.543704	-2.452541	1.155405
H	-0.805419	-2.602180	0.899986
H	-1.415832	-1.415483	2.057669
H	-3.756225	-0.353866	0.494834
H	-2.590766	0.671390	1.304698
H	-2.983666	0.900637	-0.446479
C	3.196670	-0.947297	-0.812459
C	2.212857	1.230548	-0.102034
C	2.177114	-0.714561	1.464019
H	3.248525	1.544993	0.060834
H	1.912355	1.617819	-1.083879
H	1.646297	1.733640	0.697649
H	4.177416	-0.709920	-0.386930
H	3.090568	-2.037039	-0.817566
H	3.189678	-0.608047	-1.853708
H	3.082852	-0.310923	1.930268
H	1.307285	-0.360185	2.025719
H	2.212568	-1.804774	1.555997

	47		
C4			
Ca	-0.472179	-0.006499	-1.240163
N	1.349306	0.548906	-0.096087
H	-0.581129	-0.371263	-3.264397
C	2.177917	-0.286896	0.744346
C	1.682188	1.972560	-0.075006
C	3.061699	2.272902	-0.699806
C	0.629178	2.700106	-0.927298
C	1.634083	2.574904	1.344798
H	3.285626	3.346141	-0.688709
H	3.863413	1.767860	-0.154119
H	3.088770	1.922410	-1.735428
H	0.821112	3.774957	-0.986659
H	0.627066	2.326835	-1.962276
H	-0.375414	2.593327	-0.487379
H	1.837158	3.652236	1.337708
H	0.647170	2.411637	1.790797
H	2.375335	2.104708	1.997109
C	2.549677	-1.666558	0.140762

H	-3.345570	-0.272135	3.552504
H	-1.908253	1.565183	2.887787
Si	-2.198665	0.122567	2.688375
H	-1.022777	-0.713818	3.040058
C	-2.665687	-0.158360	0.878263
C	-2.674202	-1.450633	0.320568
C	-3.082953	0.906619	0.059983
C	-3.058754	-1.666125	-1.002176
C	-3.470201	0.694095	-1.263612
C	-3.443545	-0.591434	-1.804851
H	-3.708072	-0.753380	-2.842379
H	-3.773130	1.533443	-1.880754
H	-3.102946	1.916501	0.459922
H	-3.037559	-2.668216	-1.416930
H	-2.372938	-2.302051	0.924824
H	3.136681	0.194791	0.997874
H	1.703029	-0.495950	1.726168
C	3.213819	-2.503404	1.240922
C	1.296880	-2.405503	-0.353053
C	3.518687	-1.495195	-1.032362
H	0.480022	-2.348546	0.379342
H	0.966847	-2.029497	-1.332422
H	1.503342	-3.468197	-0.518780
H	4.086360	-1.984751	1.652023
H	2.522322	-2.696040	2.068894
H	3.553226	-3.470063	0.853568
H	4.470704	-1.074280	-0.692428
H	3.731571	-2.454540	-1.517268
H	3.095073	-0.818059	-1.778883

	47		
C5*			
Ca	1.150213	-0.432354	2.166672
N	1.050791	-0.357980	-0.319484
H	2.826905	0.116233	3.232337
C	0.786848	0.960904	-0.974600
C	2.415824	-0.964811	-0.670351
C	3.551974	0.043025	-0.452161
C	2.693667	-2.145657	0.280143
C	2.465903	-1.457905	-2.124063
H	4.502506	-0.471567	-0.619570
H	3.519809	0.886293	-1.144633
H	3.572694	0.423146	0.573771
H	3.510733	-2.750506	-0.121129
H	3.054362	-1.805680	1.262861
H	1.831514	-2.801292	0.416379
H	3.483488	-1.775291	-2.369887
H	1.804245	-2.311310	-2.291835
H	2.183288	-0.669490	-2.827071
C	0.550680	2.216644	-0.092867
H	-0.406574	-1.339848	-2.245630
H	-0.074741	-2.881017	-0.452669
Si	-0.357950	-1.462840	-0.751358
H	-0.214163	-1.960417	1.970252
C	-2.090330	-0.867549	-0.298309
C	-2.823082	-0.222392	-1.308304
C	-2.738314	-1.123048	0.918713
C	-4.137949	0.185622	-1.101946

C	-4.056560	-0.727126	1.123019
C	-4.756695	-0.063423	0.119019
H	-5.783797	0.247958	0.283008
H	-4.540954	-0.941429	2.070907
H	-2.182996	-1.634187	1.700485
H	-4.680871	0.686977	-1.897257
H	-2.365407	-0.047124	-2.278423
H	1.604991	1.189756	-1.662246
H	-0.098534	0.873554	-1.618660
C	0.266123	3.365366	-1.076535
C	-0.682836	2.065326	0.806527
C	1.770207	2.617767	0.746485
H	-1.593511	1.928479	0.219320
H	-0.651562	1.200062	1.481383
H	-0.808154	2.952777	1.434009
H	1.121872	3.543849	-1.735670
H	-0.604867	3.148461	-1.702903
H	0.064728	4.294605	-0.535478
H	2.644248	2.801518	0.116414
H	1.555958	3.543447	1.289867
H	2.083538	1.888272	1.505406

47

C6

Ca	1.086958	-0.528284	2.208288
N	1.018010	-0.352843	-0.362540
H	2.818680	0.194820	3.053153
C	0.768549	0.997642	-0.968845
C	2.412613	-0.928994	-0.667963
C	3.521798	0.092917	-0.392701
C	2.677555	-2.120013	0.273883
C	2.522908	-1.396570	-2.126924
H	4.484671	-0.405757	-0.537133
H	3.500099	0.945954	-1.073394
H	3.499386	0.450496	0.641206
H	3.501157	-2.720596	-0.120106
H	3.025272	-1.786465	1.262760
H	1.819769	-2.786827	0.387343
H	3.555845	-1.682629	-2.344304
H	1.893620	-2.267037	-2.332575
H	2.243492	-0.603784	-2.826597
C	0.517427	2.230722	-0.059280
H	-0.350648	-1.356353	-2.429724
H	-0.077451	-2.810454	-0.498078
Si	-0.339662	-1.421431	-0.940081
H	-0.241938	-2.016377	2.718173
C	-2.048729	-0.860527	-0.417133
C	-2.861290	-0.208327	-1.358037
C	-2.590363	-1.151438	0.844456
C	-4.163242	0.168650	-1.043456
C	-3.892847	-0.773851	1.157771
C	-4.678607	-0.110553	0.219223
H	-5.695077	0.179444	0.466888
H	-4.296112	-1.005780	2.138605
H	-1.980952	-1.664673	1.589190
H	-4.776563	0.672037	-1.784238
H	-2.479137	-0.000750	-2.354440
H	1.596006	1.242639	-1.639445

H	-0.110160	0.936469	-1.626726
C	0.227152	3.393590	-1.025286
C	-0.719442	2.052396	0.829153
C	1.726469	2.624309	0.796659
H	-1.621934	1.880424	0.238486
H	-0.660551	1.205548	1.524077
H	-0.874753	2.945063	1.442106
H	1.080144	3.583783	-1.684813
H	-0.647108	3.188073	-1.651452
H	0.028633	4.313407	-0.467685
H	2.599599	2.842108	0.175763
H	1.494105	3.530780	1.364686
H	2.042219	1.872338	1.532310

44

C7

N	-0.936737	0.583197	-0.288902
C	-2.140755	-0.186554	-0.646081
C	-1.168756	1.915553	0.351674
C	-2.022408	1.793383	1.621005
C	0.178321	2.513584	0.775924
C	-1.855098	2.880272	-0.632205
H	-2.142588	2.782538	2.073013
H	-3.024243	1.407145	1.421528
H	-1.541984	1.140660	2.352752
H	0.013048	3.478775	1.262696
H	0.696327	1.859318	1.480803
H	0.841101	2.696789	-0.075078
H	-2.019756	3.858331	-0.168752
H	-1.238752	3.027363	-1.523667
H	-2.829034	2.499415	-0.952783
C	-2.307289	-1.606690	-0.039185
H	0.107496	-0.681967	-2.301269
H	0.783978	1.603893	-2.133211
Si	0.461893	0.383778	-1.326478
C	2.056035	-0.083588	-0.436784
C	3.285642	0.164983	-1.061520
C	2.070206	-0.712410	0.813871
C	4.485783	-0.208181	-0.463887
C	3.267510	-1.084359	1.418788
C	4.478175	-0.834275	0.779348
H	5.412928	-1.123296	1.249466
H	3.256394	-1.568741	2.390448
H	1.131611	-0.905448	1.325152
H	5.427302	-0.005532	-0.965047
H	3.310674	0.663066	-2.028096
H	-3.021189	0.392482	-0.353305
H	-2.220094	-0.298230	-1.737239
C	-3.680103	-2.117358	-0.500300
C	-1.231161	-2.569996	-0.551228
C	-2.267862	-1.581424	1.490231
H	-1.242783	-2.641930	-1.643033
H	-0.226926	-2.264038	-0.244746
H	-1.400946	-3.574016	-0.147799
H	-4.488868	-1.475271	-0.134471
H	-3.746389	-2.151315	-1.593262
H	-3.863867	-3.129226	-0.125615
H	-3.081301	-0.979202	1.905656

H	-2.368372	-2.595932	1.889993
H	-1.322559	-1.168533	1.850967

References

[S1] Hasegawa, A., Naganawa, Y., Fushimi, M., Ishihara, K., Yamamoto, H. *Org. Lett.*, **2006**, 8, 3175–3178.

[S2] Cattoën, X., Solé, S., Pradel, C., Gorntzka, H., Miqueu, K., Bourissou, D. & Bertrand, G. *J. Org. Chem.*, **2003**, 68, 911–914.

[S3] Tussing, S., Kaupmees, K. & Paradies, J. *Chem. Eur. J.*, **2016**, 22, 7422–7426.

[S4] José Barluenga, Agustín Jiménez-Aquino, Fernando Aznar, and Carlos Valdés, *J. Am. Chem. Soc*. **2009**, 131, 4031-4041.

[S5] Goki Hirata, Naoshi Yamada, Shohei Sanada, Gen Onodera, Masanari Kimura *Org. Lett.* **2015**, 17, 600–603.

[S6] R. Verhé, N. De Kimpe, L. De Buyck, *Tetrahedron* **1979**, *36*, 131–142.

[S7] R. Wang, M. Ma, X. Gong, G. B. Panetti, X. Fan, P. J. Walsh, *Org. Lett*. **2018**, 20, 2433-2436

[S8] O. V. Dolomanov, L. J. Bourhis, R. J. Gildea, J. A. K. Howard, H. Puschmann, *J. Appl. Cryst.* **2009**, *42*, 339-341.

[S9] G. M. Sheldrick, *Acta Cryst.* **2015**, *A71*, 3-8.

[S10] G. M. Sheldrick, *Acta Cryst.* **2008**, *A64*, 112-122.

[S11] M. J. Frisch, G. W. Trucks, H. B. Schlegel, G. E. Scuseria, M. A. Robb, J. R. Cheeseman, G. Scalmani, V. Barone, B. Mennucci, G. A. Petersson, H. Nakatsuji, M. Caricato, X. Li, P. H. Hratchian, A. F. Izmaylof, J. Bloino, G. Zheng, J. L. Sonnenberg, M. Hada, M. Ehara, K. Toyota, R. Fukuda, J. Hasegawa, M. Ishida, T. Makajima, Y. Honda, O. Kitao, H. Nakai, T. Vreven, J. A. Montgomery, J. E. Peralta, F. Ogilaro, M. Bearpark, J. J. Heyd, E. Brothers, K. N. Kudin, V. N. Staroverov, T. Keith, R. Kobayashi, J. Normand, K. Raghavachari, A. Rendell, J. C. Burant, S. S. Iyengar, J. Tomasi, M. Cossi, N. Rega, J. M. Millam, M. Klene, J. E. Knox, J. E. Cross, V. Bakken, C. Adamo, J. Jaramillo, R. Gomperts, R. E. Stratmann, O. Yazyev, A. J. Austin, R. Cammi, C. Pomelli, J. W. Ochterski, R. L. Martin, K. Morokuma, V. G. Zakrezewski, G. A. Voth, P. Salvador, J. J. Dannenberg, S. Dapprich, A. D. Daniels, O. Farkas, J. B. Foresman, J. V. Ortiz, J. Cioslowski, D. J. Fox, Gaussian 09 Rev. D. Wallingford CT, **2013**

[S12] A. D. Becke, J. Chem. Phys. **1993**, 98, 1372–1377.

[S13] J. P. Perdew, Electronic Structure of Solids, Akademie Verlang, Berlin, **1991**.

[S14] W. J. Hehre, L. Radom, P. v. R. Schleyer, J. A. Pople, *Ab Initio Molecular Orbital Theory*, John Wiley, New York, **1986**.

[S15] T. Clark, J. Chandrasekhar, G. W. Spitznagel, P. v. R. Schleyer, J. Comp. Chem. **1983**, 4, 294–301.

[S16] G. Scalmani, M. J. Frisch, *J. Chem. Phys.* **2010**, *132*, 114110-1–16.

[S17] N. van Eikema Hommes, Molecule V2.3, Erlangen, **2016**
